# Unified Method for Target and Non-Target Monitoring of Pesticide Residues in Fruits and Fruit Juices by Gas Chromatography-High Resolution Mass Spectrometry

**DOI:** 10.3390/foods12040739

**Published:** 2023-02-08

**Authors:** Mireya Granados-Povedano, Irene Domínguez, Francisco Egea-González, Antonia Garrido Frenich, Francisco Javier Arrebola

**Affiliations:** Department of Chemistry and Physics (Analytical Chemistry Area), Research Centre for Mediterranean Intensive Agrosystems and Agri-Food Biotechnology (CIAIMBITAL), Agrifood Campus of International Excellence ceiA3, University of Almeria, E-04120 Almeria, Spain

**Keywords:** GC-Q-Exactive Orbitrap MS, juices, fruits, ultra-trace analysis, pesticide residues, retrospective analysis

## Abstract

A new polyvalent wide-scope analytical method, valid for both raw and processed (juices) fruits, combining target and non-target strategies, has been developed and validated to determine low concentrations of 260 pesticides, as well as many potential non-target substances and metabolites. The target approach has been validated according to SANTE Guide requirements. Trueness, precision, linearity, and robustness values were validated in raw fruit (apple) and juice (apple juice) as representative solid and liquid food commodities. Recoveries were between 70–120% and two ranges of linearity were observed: 0.5–20 μg kg^−1^ (0.5–20 μg L^−1^ apple juice) and 20–100 μg kg^−1^ (20–100 μg L^−1^ apple juice). The limits of quantification (LOQs) reached were lower than 0.2 μg kg^−1^ in apple (0.2 μg L^−1^ apple juice) in most cases. The developed method, based on QuEChERS extraction followed by gas chromatography-high resolution mass spectrometry (GC-HRMS), achieves part-per-trillions lower limits, which allowed the detection of 18 pesticides in commercial samples. The non-target approach is based on a retrospective analysis of suspect compounds, which has been optimized to detect up to 25 additional compounds, increasing the scope of the method. This made it possible to confirm the presence of two pesticide metabolites which were not considered in the target screening, phtamlimide and tetrahydrophthalimide.

## 1. Introduction

Food safety is essential for the development and proper functioning of the body. Raw and processed (juices) fruits are largely consumed by the general population. Multi-purpose and wide-ranging analytical methodologies are highly demanded to routinely monitor pesticide residues potentially present in such agricultural commodities, particularly when considering the youngest in the population [1]. Children are within the largest consumers of fruits and vegetables, a stage of age that is accompanied by a high intake of juice, a processed product that can be contaminated by a variety of pesticide residues [2]. Although risks to consumers from the presence of pesticide residues in food are currently estimated substance by substance, the European Food Safety Authority (EFSA) considers the fact that a number of pesticides have similar effects and their impact on human health could be greater in combination than individually; therefore, EU regulations on pesticides in food and feed stipulate that the cumulative and synergistic effects of pesticides should be considered for dietary risk assessment. They also state that residues of pesticides should not have any harmful effects on human health, taking into account known cumulative and synergistic effects.

In many cases, for industrial or domestic production of fruit juices, the whole fruit is used, including the skin, where several pesticide residues are potentially located [3]. This is especially frequent in juices obtained from thin-skinned fruits, such as pear, apple, grape, peach or similar, or the case of citrus. These types of agricultural products are particularly sensitive to insect and fungal pests and therefore need to be treated frequently with different types of plant protection products to avoid a loss of production, and sometimes even in post-harvest treatments to extend their shelf life. In fact, some of these commodities are among the fresh products where most residues are detected in control programs. Regardless of whether they are quantified above or below their individual MRL, the additional problem is that in these types of fruit, a large variety of active substances can be detected in the same sample, which is of particular interest due to likely cumulative or synergistic effects. 

Of special concern is the limited information focused on pesticide residues in food commodities prevalent in the children´s population group. However, there is available information on neurological and immunological damage and cancer in children of all ages, including fetuses, which in addition shows the vulnerability of pregnant women to pesticide exposure [4,5].

In consequence, the determination of low concentrations of pesticide residues, even at trace levels, is key. For this, sensitive and reliable methods are required with a larger scope in terms of number of pesticides and food commodities controlled. To achieve these goals, progress in analytical instrumentation is proving a necessary factor. In recent years, the rise in popularity of high-resolution mass spectrometry (HRMS) for routine analysis is visible, due to the improvements of the shortcomings of this instrumentation. The advances in sensitivity, enhanced resolution power, high selectivity, and the possibility of using it for retrospective analysis [6,7] make HRMS equipment a worthy rival of the traditional triple quadrupoles, not only for the analysis of pesticides [8,9]. 

The study of pesticide residues in fruit has been covered by LC-HRMS [10,11,12] and GC-HRMS [13,14]. Although with fewer scientific studies, pesticide residues have also been determined in juice by LC-HRMS [15,16] and GC-HRMS [17,18], with the latter being two of the only works found in which GC-HRMS is used for the analysis of pesticides in juice. There are previous studies proposing analytical methods for determining pesticides in both fruit (solid samples) and juices (liquid samples) based on liquid chromatography with diode array detection (LC-DAD) [19], GC-MS/MS with triple quadrupole [20], and GC with electron capture detector (ECD) [21], namely using classical detection systems and complex extraction methods. The use of GC-HRMS with the Q-Exactive Orbitrap analyzer can improve this type of analysis, increasing reliability in the confirmation of detected pesticides and improving its sensitivity to ng kg^−1^ or ng L^−1^ levels [22]. This improvement in the sensitivity and selectivity of HRMS with respect to classical detectors reduces the risk of false-positive and -negative results [23]. To our knowledge, the application of GC-HRMS in the determination of a high number of pesticide residues simultaneously in raw fruits and fruit juices has not been reported.

Regarding the extraction methods of pesticides in fruits, there is a wide range of techniques (liquid–liquid extraction (LLE), solid phase extraction (SPE), solid phase microextraction (SPME), matrix solid phase dispersion (MSPD), gel permeation chromatography (GPC), among others) that can vary according to the analyte or matrix of interest [24]. Although there is no standardized extraction method accepted in all laboratories, lately QuEChERS and its different variations have become the preferred approach for extracting pesticides from fruits [25]. For juice samples, several extraction techniques have been proposed, such as SPE [26], dispersive liquid–liquid microextraction (DLLME) [27,28], or continuous sample drop flow microextraction (CSDF-ME) [29]. Nevertheless, to increase efficiency and operability in routine laboratories, harmonization of the methods will improve throughput. Furthermore, for juice extraction, the application of the QuEChERS procedure will also simplify extraction protocols in pesticide residue laboratories.

Retrospective analysis is another additional advantage of using GC-Q-Exactive Orbitrap MS due to the high sensitivity achieved in full scan mode [30]. This means that non-target analysis can be performed and thus monitor, in theory, an unlimited number of compounds [31]. 

Therefore, the objective of this work has been to optimize and validate an analytical method based on GC-HRMS which allows for high sensitivity and a wide scope determination of pesticides, which is applicable to the control of pesticide content in fruits and juices at levels that meet the safety requirements of vulnerable population groups, such as children, infants, and pregnant women. To achieve these objectives, a large number of target pesticides have been included together with the applicability of a retrospective analysis approach, which allows the identification of other currently used pesticides as well as transformation products and/or metabolites derived from the parent pesticides. 

## 2. Materials and Methods

### 2.1. Materials and Reagents

Pesticide standards used in this work came from Dr. Ehrenstorfer (Augsburg, Germany) or Sigma Aldrich (St. Louis, MO, USA) and were stored at −20 °C for their conservation. Purities ranged between 96 and 99% for the different standards. Sodium chloride (NaCl), magnesium sulfate (MgSO_4_), and primary secondary amine (PSA) were purchased from Scharlab (Barcelona, Spain). Dehydrated trisodium citrate (C_6_H_9_Na_3_O_9_) was acquired from Panreac AppliChem (Barcelona, Spain) and sesquihydrate dibasic sodium citrate (C_12_H_18_N0_4_O_17_) from Sigma Aldrich. Acetonitrile (gradient grade) from Honeywell (Seelze, Germany) and ethyl acetate (PAR grade) from Fluka (St. Louis, MO, USA) were used. For each pesticide, a stock solution in acetone at a concentration of 1000 mg L^−1^ was prepared and stored in amber glass vials with a screw cap. For calibration, three mix standards were prepared in ethyl acetate from the pesticide stock solutions. Propoxur-d7 was used as an internal standard at a final concentration of 25 μg kg^−1^ (25 μg L^−1^ for juices). All prepared solutions were stored at −20 °C.

### 2.2. Extraction Procedure

All fruits (apple, orange, clementine, banana, and lemon), as well as fruit juice samples (apple, orange, berries, mango, passion fruit, pineapple and pear, peach, and multi-fruits) were purchased from supermarkets in the Almeria area (Spain). Raw fruits (including peel) were first sliced following the indications of Directive 2002/63/EC31 and then homogenized. The method used for extraction of the pesticide residues was based on a QuEChERS modification previously developed by our research team [6]. An amount of 10 g of sample, or 10 mL in the case of juice, was poured into a 50 mL polypropylene centrifuge tube. Extraction was carried out using 10 mL of acetonitrile and shaking by vortex for 2 min. Next, 4 g of magnesium sulfate, 1 g of sodium chloride, 1 g of dehydrated trisodium citrate, and 0.5 g of sesquihydrate dibasic sodium citrate were added and the suspension was mixed using vortex by 2 min and later centrifuged for 6 min at 4000 rpm. For a clean-up step, 5 mL of the extract was transferred to a 15 mL centrifuge tube where 750 mg of magnesium sulfate and 125 mg of PSA were added. The tube was vortexed for 1 min and centrifuged for 6 min at 4000 rpm. Then, 1 mL of the clean extract was transferred to a chromatographic vial and the solvent evaporated under a soft stream of nitrogen. Finally, the sample extract was redissolved with ethyl acetate (950 μL) and 50 μL of the IS solution, ready for its analysis by GC-HRMS.

### 2.3. GC-Q-Exactive Orbitrap MS Parameters

The analysis was carried out by coupling a gas chromatograph (TRACE 1300) to a Q-Exactive-Orbitrap mass analyzer, in addition to a TriPlus RSH autosampler (Thermo Fisher Scientific, Bremen, Germany). For the analysis of samples, 1 µL was injected at 280 °C in splitless mode, with a splitless time of 1 min, in a cone-lined injector (78.5 mm × 4 mm ID) (Thermo Fisher Scientific). The carrier gas flow (Helium 99.999%) was 1 mL min^−1^ and using a Zebron™ ZB-5MSPLUS™ column (Phenomenex, Aschaffenburg, Germany) of 30 m length × 0.25 mm internal diameter, 0.25 μm film thickness. The oven program started at 50 °C (hold 1 min) and then increased at 20 °C min^−1^ up to 170 °C. After that, temperature was raised to 310 °C at 10 °C min^−1^ (hold 8 min). It took a total analysis time of 29 min. The type of ionization used by Q-Exactive-Orbitrap MS was electron ionization (EI) with full scan acquisition mode. A 50 µA emission current was applied to the ionization filament for generating electrons at 70 eV at a temperature of the ion source of 250 °C as well as the GC-MS transfer line. The first 5 min of the analysis was set for the filament delay. The applied scan range was 40 to 500 *m*/*z* with 1 μscan maximum injection time. Thanks to HRMS, a resolution power of 60.000 full width at half maximum (FWHM) and a 1e^6^ ions automatic gain control (AGC) target was setup.

### 2.4. Data Processing

Data analysis was carried out by Xcalibur (version 4.3.73) and TraceFinder 4.1 software (Thermo Fisher Scientific, Les Ulis, France). Xcalibur allowed for monitoring ions with an accuracy of 5 decimal places. Similarly, criteria were established to consider that a compound was present in a sample when a mass error ≤ 5 ppm was observed for the base peak and two other confirmation peaks to increase the reliability of the results. Through the use of Xcalibur and other tools, such as the NIST standard reference database, a proprietary database with a total number of 260 compounds was created (Table S1 of Supplementary Material). In addition, after a bibliographic search, an additional database of 25 suspected compounds was created to increase the given scope of the analytical method with the possibility of retrospective analyses. The suspected strategy considers solid bibliographic information and the results obtained in the target analyses, which leads us to think about the likely presence of additional non-target compounds such as metabolites of detected pesticides. These compounds are then included in retrospective analysis.

### 2.5. Method Validation

Apple and apple juice were selected as representative matrices to validate the method for raw fruits and juices, respectively. Linearity, trueness, and precision were evaluated following the established recommendations of the DG SANTE [32]. Calibration curves were studied in triplicate for determining linearity (0.5, 1, 5, 10, 20, 50, 100 μg kg^−1^, or μg L^−1^ for juices). The working range was considered as linear when recoveries of individual standards were between 70 and 120%, a regression coefficient (R^2^) higher than 0.98 was observed, and adequate precision was achieved (<20%). Precision was studied by calculating the relative standard deviation (RSD %) of blank samples spiked (intraday *n* = 5; interday *n* = 5 days) at two different concentrations, 1 and 10 μg kg^−1^, (μg L^−1^ for juices). The recoveries of pesticides at 1 and 10 μg kg^−1^ (μg L^−1^ for juices) were studied for determining the trueness. LOQs were determined following two different approaches recommended for HRMS analyzers: (i) 10 times the standard deviations obtained for the lowest level of calibration curve [6], and (ii) the lowest concentration level offering RSD values ≤ 20% and acceptable recoveries (values ranging from 70 to 120%). LODs were determined similarly to the first LOQ calculation, but considering 3 times standard deviation [33].

## 3. Results and Discussion

### 3.1. Validation Data

In this study, the validation of 260 target compounds was carried out and the results are shown in Tables S2 and S3 of the Supplementary Material. Two different linear ranges could be considered in the range of concentrations studied (between 0.5 and 100 μg kg^−1^, μg L^−1^ juices). For quantifying pesticide residues at very low concentrations, a calibration range between 0.5–20 μg kg^−1^ (0.5–20 μg L^−1^ juices) was proposed. Approximately 70% of the studied pesticides could reach such a low concentration level. For the other 30% of compounds their calibration range was between 1 and 20 μg kg^−1^ (1–20 μg L^−1^ juices). The only exceptions were acrinathrin, difenoconazole, dimethomorph, diniconazole, ethion, feniamiphos, fenamiphos sulfoxide, tolylfluanid, and transfluthrin in juices that presented an LOQ of 5.0 μg L^−1^. A second calibration range was proposed for quantifying higher concentrations in the range 20–100 μg kg^−1^ (20–100 μg L^−1^ juices) suitable for the routine analysis of the studied pesticides at low–medium concentrations. As can be seen in Table S2, the determination coefficients (R^2^) were 0.99 in most compounds in both apple and apple juice samples with a few exceptions that were always ≥0.98.

In most cases, recovery ranged between 70 and 120%. It should be noted that 94% of the compounds showed an optimal recovery at 1 μg kg^−1^. Just in the case of alpha-endosulfan, beta-endosulfan, beta-hexachlorocyclohexane, delta-hexachlorocyclohexane-and o,p′-DDE in apple, low recovery values were observed. Otherwise, for apple juice, acrinathrin was the only compound whose recovery values were not adequate, with values below 50%.

For apple and apple juice, interday precision was slightly higher than intraday precision, as might be expected. Comparing matrices, precision (intraday and interday) for apple was lower than for apple juice. In general, the validation showed good results (RSD < 20%) for 255, that is, 98 % of the total target compounds.

If LOQ is considered as the lowest values of the calibration curve where RSD ≤ 20%, LOQ was established at 0.5 μg kg^−1^ in the case of apple and at 0.5 μg L^−1^ in the case of apple juice (over 70% of the studied compounds). For the remaining percentage of compounds, the LOQ was set at 1 μg kg^−1^. Considering the alternative criteria for calculating LOQs, those values were significantly lower, ranging between 0.02 and 0.60 μg kg^−1^ for apple and between 0.05 and 0.90 μg L^−1^ for apple juice. The LOQs found in the literature are usually between 1 and 10 μg kg^−1^ (μg L^−1^) [6,34,35], higher than those achieved by the proposed method.

### 3.2. Application of the Method to Commercial Samples

Different samples randomly acquired in local shops were studied to verify the applicability of the validated method. A total of 83 fruit samples were analyzed, orange (*n* = 40), banana (*n* = 6), apple (*n* = 8), clementine (*n* = 25), and lemon (*n* = 4). Additionally, 15 different juice samples were also included in the study: multi-fruit (*n* = 7), orange (*n* = 2), apple (*n* = 1), berries (*n* = 1), mango (*n* = 1), passion fruit (*n* = 1), pineapple-pear (*n* = 1), and peach (*n* = 1) juices.

As the internal quality control and in order to check the stability of the method, each batch of analyzed samples included the analysis of two blanks and two blanks spiked at 1 μg kg^−1^ (μg L^−1^) and 10 μg kg^−1^ (μg L^−1^) and two calibration curves. Blind and duplicated samples were also analyzed. Stability of the retention time of the analytes was also evaluated. In this way, recovery rates were checked, and the presence of cross contamination was discarded. The pesticide residues found in the studied samples can be seen in Table 1. The results were established according to the Maximum Residue Level (MRL) found in the European Union database [36]. In the case of juices, Reg. 396/2005: Article 20 establishes that if there is no specific MRL for the processed product, that of the raw product will be used, or failing that, it will be 0.01 mg kg^−1^. For those samples containing concentrations of pesticide residues exceeding the established MRL, another independent extraction of the same commercial sample was performed for confirmation of the results.

The results have shown that 70% of the total fruit samples analyzed contained pesticide residues. Of these, 33% of the studied fruit samples contained only one active substance residue, while 67% contained two or more different active substances. Notably 13% of the total analyzed samples contained pesticide residues above the regulated MRL established by the European Union [37]. A total of 18 different pesticides were detected in the studied samples, 55% of them were insecticides, 40% fungicides, and finally 5% herbicides. Pyrymethanil and pyriproxyfen were the most detected pesticides in the fruit samples. Different pesticides were present at concentrations above the established MRLs, such as the case of metalaxyl (apple), chlorpyrifos methyl (orange), chlorpyrifos ethyl (banana), and ethoxyquin (lemon).

The results on market samples, where the presence of multiple active substances is detected in two thirds of the samples analyzed, confirm the concern of European authorities about cumulative exposure and therefore the need for analytical methods, such as the one developed here, capable of detecting a wide range of active substances in different matrices at low concentrations, even below MRLs in potentially toxic metabolites that are outside the scope of analytical methods based on target compound analysis.

In the case of juices, data are summarized in Table 2. All the studied samples presented at least one pesticide residue above the LOQ of the method. Nine of them contained one compound exceeding the regulated MRL and in another four samples, two compounds exceeded these limits. A total of 15 different pesticides were observed in the juices, of which 53% were fungicides and 47% were insecticides. Thanks to the proposed method, it has been possible to detect different pesticide residues in juice, such as dieldrin (present at high concentrations in 13 of the studied samples), fludioxonil, or pyrimethanil. The presence of dieldrin and aldrin in some of the samples is of high concern since they are banned pesticides [38,39]. 

If the results obtained in fruit and juice are percentage-compared, the number of pesticide residues detected in fruit was slightly higher than in the juices. These detected pesticides were different, except for 2-phenylphenol, chlorpyrifos ethyl, pyrimethanil, and tebuconazole that appear in both types of samples. As a significant difference, in fruits, the most detected pesticide residues were insecticides, while in fruit juices fungicides were the majority.

### 3.3. Suspect Analysis

Through an exhaustive bibliographic search, it was possible to detect possible additional compounds that could be present in the matrices studied. A list of candidates was created using specialized software (TraceFinder and Xcalibur), and the NIST database. They were searched in the already obtained chromatograms. This was possible thanks to the non-target acquisition mode used for this study, a broad-spectrum full scan mode not focused on just a few fragments. Table S4 of the Supplementary Material lists the 25 suspected compounds found in the bibliography with their highest intensity *m*/*z*. The selected compounds were detected both in fruits and in juice, following the line of work of this study. 

**Figure 1:**
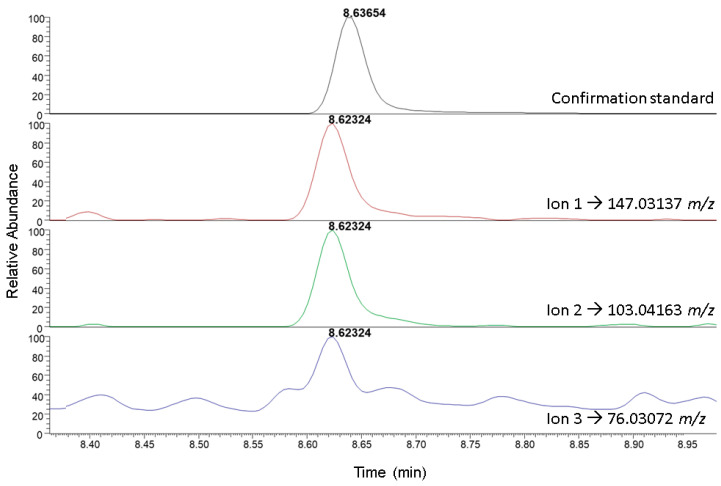
Extracted ion chromatograms of phthalimide in a sample of orange juice and the confirmation standard used.

The search revealed the presence of two candidate compounds in the studied matrices, as indicated in Table 3. For a higher reliability, both compounds, phthalimide and tetrahydrophthalimide, were confirmed using analytical standards (Figure 1). They were also validated in both apple and apple juice. For this, the same criteria were followed as for the 260 target compounds, whose validation values are shown in Tables S5 and S6 in the Supplementary Material. Both compounds manifested acceptable linearities with R^2^ values of 0.99. Phthalimide recoveries were slightly higher in both apple and juice compared to tetrahydrophthalimide, while the limit of quantification was lower for phthalimide. On the other hand, the precision was very similar for both metabolites regardless of the matrix. 

Phthalimide and tetrahydrophthalimide are considered metabolites of the pesticides folpet and captan or captafol, respectively [35]. Folpet and captan were also searched as shown in Table S4 of the Supplementary Material. Captan and folpet can give rise to their metabolites in different ways: exposure to natural light, increase in the temperature, hydrolysis in a basic medium, and some extraction or detection processes [40,41]. In the case of fruit, phthalimide was only detected in orange and banana at a very low concentration with respect to its MRL. It should be noted that in orange the presence of phosmet was observed, an insecticide whose degradation can also lead to phthalimide [35]. Therefore, this could explain the presence of phthalimide in this orange sample.

On the contrary, in juices, both phthalimide and tetrahydrophthalimide were detected in different fruit juices (multi-fruits, orange, apple, berries, mango, pineapple and pear, passion fruit, and peach). In some cases, such as multifruit juices, the two metabolites were monitored in the same sample. In addition to this, in juice it was observed that these compounds exceeded their established MRL in three samples. Tetrahydrophthalimide exceeded the legal limit in peach and pineapple and pear juices. Phthalimide was found in orange juice at a value twice that allowed for raw fruit. It is important to be able to find these types of compounds in the analysis of samples, since the metabolites can be more harmful to health than their parent pesticides [42].

## 4. Conclusions

The use of GC-HRMS has proven to be a good tool for a routine multi-residue analysis. GC-Q Exactive Orbitrap MS together with a modified QuEChERS has allowed the detection of different pesticides in both solid and liquid foodstuff samples (raw fruits and juices). The analytical method has been validated and developed for a total of 260 pesticides of different families and types (insecticides, fungicides, and herbicides) in apple and apple juice (selected as representative matrices). In most compounds, the LOQs could reach ultra-trace levels, less than 0.5 μg kg^−1^ (μg L^−1^) in several cases, which demonstrates the improved sensitivity of the methodology used. 

The method was successfully applied to the analysis of 83 different fruit samples where a total of 18 different pesticides were detected in 70% of them. On the other hand, 15 juices were also analyzed, finding 15 different pesticides. In total, 100% of the tested samples contained at least one of those pesticide residues. Additionally, the proposed analytical methodology, which combines the target with a retrospective analysis of non-target compounds, achieves the identification of toxic substances that escape the usual food safety analytical controls, as demonstrated by the detection of two phthalimide metabolites at levels well above the permitted levels. Considering that re-analysis of samples is not necessary, this HRMS-based methodology can potentially be a reliable source of information in the field of risks assessment.

## Figures and Tables

**Table 1:** Pesticide residues detected in fruit samples.

Matrix	Pesticide	Detection Frequency	Pesticide Type *	Concentration Range (μg kg^−1^)	MRL EU (μg kg^−1^)
Apple	Metalaxyl	1	F	12.4	10
	Pyrimethanil	3	F	0.6–84.3	15,000
	Pyriproxyfen	3	I	4.5–35	700
Orange	2-Phenylphenol	8	F	3.1–494.4	10,000
	Chlorpyrifos ethyl	1	I	8.1	10
	Chlorpyrifos methyl	11	I	2.4–57.5	10
	Fluvalinate	1	I	78.1	400
	Metalaxyl	2	F	2.3–15.7	700
	Oxyfluorfen	1	H	9.1	50
	Phosmet	2	I	6–73.1	500
	Propiconazole	1	F	12.3	9000
	Pyrimethanil	19	F	1.6–1519.8	8000
	Pyriproxyfen	25	I	2.6–27.9	600
	Tebuconazole	1	F	3	900
Clementine	Chlorpyrifos methyl	2	I	1.6–3.3	10
	Deltamethrin	2	I	17.9–25.4	40
	Pyridaben	1	I	33.5	300
	Pyrimethanil	6	F	1.2–111.1	8000
	Pyriproxyfen	5	I	4.1–37.4	600
	Tetraconazole	2	F	3.4–3.5	20
Banana	Azoxystrobin	1	F	47.1	2000
	Bifenthrin	2	I	7.6–8.3	100
	Chlorpyrifos ethyl	6	I	4.2–17.6	10
	Pyrimethanil	3	F	1.3–7.4	15,000
	Tebuconazole	1	F	1.3	300
Lemon	Ethoxyquin	3	I	20.2–50.6	50
	Pyriproxyfen	1	I	62.8	600

* I, insecticide; F, fungicide; H, herbicide.

**Table 2:** Pesticide residues detected in juice samples.

Matrix	Pesticide	Detection Frequency	Pesticide Type *	Concentration Range (μg L^−1^)	MRL EU ** (μg L^−1^)
Multi-fruits	1,4-Dimethylnaphthalene	7	I	0.1–0.3	10
	Aldrin	7	I	3.4–3.6	10
	Cypermethrin	7	I	1.3–12.6	10
	Dieldrin	7	I	273.4–366.5	10
	Fludioxonil	5	F	2.5–8.3	10
	Penconazole	7	F	0.2–0.5	10
	Phenol 2,4,6-Trichloro	7	F	3.5–3.6	10
	Propiconazole	1	F	0.5	10
	Pyrimethanil	4	F	2.1–15.6	10,000
Others	1,4-Dimethylnaphthalene	4	I	0.2–2.3	10
	2-Phenylphenol	2	F	0.5–0.6	
	Aldrin	6	I	3.1–3.7	10
	Chlorpyrifos ethyl	1	I	0.5	10
	Cypermethrin	6	I	1.5–7.1	2000
	Dieldrin	8	I	2.1–370.3	10
	Fludioxonil	4	F	2.8–59.8	2000
	Penconazole	5	F	0.3–0.6	10
	Phenol 2,4,6-trichloro	7	F	3.5–3.6	10
	Prallethrin	1	I	7.4	10
	Pyrimethanil	1	F	3.4	10
	Tebuconazole	2	F	0.2–1.5	10

* I, insecticide; F, fungicide; ** maximum residue levels for the raw product as established by Reg. 396/2005: Article 20.

**Table 3:** Suspect residues detected in fruit and juice samples.

Matrix	Pesticide	Detection Frequency	Pesticide Type *	Concentration Range (μg kg^−1^ or μg L^−1^)	MRL EU ** (μg kg^−1^ or μg L^−1^)
FRUITS					
Orange	Phthalimide	6	I	1.8–7.3	30
Banana	Phthalimide	2	I	1.5–2	30
JUICES					
Multi-fruits	Tetrahydrophthalimide	4	I	0.7–5.6	10
	Phthalimide	7	F	0.7–3.4	30
Orange	Phthalimide	1	F	58.1	30
Apple	Tetrahydrophthalimide	1	I	0.9	10
	Phthalimide	1	F	0.3	30
Berries	Tetrahydrophthalimide	1	I	0.4	10
	Phthalimide	1	F	0.6	30
Mango	Phthalimide	1	F	9.9	30
Passion fruit	Phthalimide	1	F	1.1	30
Pineapple and pear	Tetrahydrophthalimide	1	I	12.1	10
	Phthalimide	1	F	6.9	30
Peach	Tetrahydrophthalimide	1	I	10.4	10
	Phthalimide	1	F	6.1	30

* I, insecticide; F, fungicide; ** maximum residue levels for the raw product as established by Reg. 396/2005: Article 20.

## Data Availability

The data presented in this study are available on request from the corresponding author.

## Supplementary Materials

The following supporting information can be downloaded at https://www.mdpi.com/article/10.3390/foods12040739/s1, Table S1: Database created with GC-Q-Exactive-Orbitrap parameters of target pesticides; Table S2: Summary of data obtained for validation tests performed to matrices studies; Table S3: Limits of detection (LODs) and quantification (LOQs) for the validation in apple and apple juice [43,44,45]. Table S4: List of suspected compounds searched by retrospective analysis; Table S5: Summary of data obtained for validation tests performed to tetrahydrophthalimide and phthalimide; Table S6: Limits of detection (LODs) and quantification (LOQs) for the validation of tetrahydrophtalimide and phthalimide in apple and apple juice.
