# Supplementary material for: Unified Method for Target and Non-Target Monitoring of Pesticide Residues in Fruits and Fruit Juices by Gas Chromatography-High Resolution Mass Spectrometry
Source: Foods. 2023 Feb 8;12(4):739. doi: 10.3390/foods12040739 (PMC9955418; doi:10.3390/foods12040739)
Supplement: Supplementary file 1 [file foods-12-00739-s001.zip › foods-2144628-supplementary.pdf]

Supplementary Material

Unified method for targeted and non-targeted monitoring of pesticide residues in fruits and fruit juices by gas chromatography-high resolution mass spectrometry

M. Granados-Povedano[1], I. Domínguez[1], F. Egea-González[1], A. Garrido Frenich[1], F.J. Arrebola[1,*]

1 *Department of Chemistry and Physics (Analytical Chemistry Area), Research Centre for Mediterranean Intensive Agrosystems and Agri-Food Biotechnology (CIAIMBITAL), Agrifood Campus of International Excellence ceiA3, University of Almeria, Almeria, E-04120, Spain;*

**Correspondence: arrebola@ual.es*

Table S1. Database created with GC-Q-Exactive-Orbitrap MS parameters of target pesticides

Compound name	Elemental Composition	Polarity	RT	Quan ion (*m*/*z*)	Confirming ion 1 (*m*/*z*)	Confirming ion 2 (*m*/*z*)
**1.4-dimethylnaphthalene**	$C_{12}H_{12}$	Positive	8,57	156,0939	141,0704	142,0733
**2-phenylphenol**	$C_{12}H_{10}O$	Positive	9,12	169,0648	170,0724	141,0699
**3.5-Dichloroaniline**	$C_6H_5C_{12}N$	Positive	8,17	160,9799	126,0111	91,04219
**4.4Dibromobenzophenone**	$C_{13}H_8Br_2O$	Positive	15,35	182,9446	154,9496	76,0313
**4.4-Dichlorobenzophenone**	$C_{13}H_8C_{12}O$	Positive	13,38	138,9945	249,9947	251,99166
**4-Chloro-3-methylphenol**	C_7H_7ClO	Positive	7,27	142,0185	107,0491	77,03854
**Aclonifen**	$C_{12}H_9ClN_2O_3$	Positive	15,79	264,0296	182,0599	194,04736
**Acrinathrin**	$C_{26}H_{21}F_6NO_5$	Positive	15,54	181,0647	208,0757	152,06213
**Alachlor**	$C_{14}H_{20}ClNO_2$	Positive	12,41	160,1119	188,1069	146,09636
**Aldrin**	$C_{12}H_8C_{16}$	Positive	13,32	262,8565	292,9268	219,98404
**Anthraquinone**	$C_{14}H_8O_2$	Positive	13,29	180,0575	76,0313	207,0446
**Atrazine**	$C_8H_{14}ClN_5$	Positive	10,98	200,0703	202,0669	173,04682
**Azoxystrobin**	$C_{22}H_{17}N_3O_5$	Positive	21,6	344,103	329,0795	388,09311
**Benalaxyl**	$C_{20}H_{23}NO_3$	Positive	16,13	148,112	176,107	206,1176
**Benfluralin**	$C_{13}H_{15}F_3N_3O_4$	Positive	10,28	292,054	264,0227	276,05894
**Benfuresate**	$C_{12}H_{16}O_4S$	Positive	12,05	163,0753	135,0805	145,06488
**Benodalil**	$C_{13}H_{10}INO$	Positive	15,89	230,9307	202,9358	76,0313
**Benoxacor**	$C_{11}H_{11}Cl_2NO_2$	Positive	11,96	120,0449	176,0712	224,04783
**Benzene. hexachloro**	C_6C_{16}	Positive	10,93	283,8096	248,8407	281,81257
**Benzyl benzoate**	$C_{14}H_{12}O_2$	Positive	11,25	105,034	91,05477	77,03912
**Bifenazato**	$C_{17}H_{20}N_2O_3$	Positive	17,28	199,0997	258,10004	184,07623
**Bifenox**	$C_{14}H_9CL_2NO_5$	Positive	17,54	340,9851	173,0153	309,96695
**Bifenthrin**	$C_{23}H_{22}ClF_3O_2$	Positive	17,18	181,1011	166,0776	165,0699
**Biphenyl**	$C_{12}H_{10}$	Positive	8,05	154,0775	152,0621	76,03075
**Bitertanol**	$C_{20}H_{23}N_3O_2$	Positive	18,94	170,0732	168,1137	280,1086
**Boscalid**	$C_{18}H_{12}Cl_2N_2O$	Positive	19,96	139,9898	342,0321	111,99485
**Bromacil**	$C_9H_{13}BrN_2O_2$	Positive	12,79	204,9613	206,9587	230,97691
**Bromocyclen**	$C_8H_5BrCl_6$	Positive	12,07	236,8408	356,7982	358,7957
**Bromophos-ethyl**	$C_{10}H_{12}BrCl_2O_3PS$	Positive	14,29	302,846	358,9085	241,87141
**Bromophos-methyl**	$C_8H_8BrCl_2O_3PS$	Positive	13,54	330,8774	315,8537	328,87957
**Bromopropylate**	$C_{17}H_{16}Br_2O_3$	Positive	17,3	340,8994	182,944	184,94196
**Bupirimate**	$C_{13}H_{24}N_4O_3S$	Positive	15	208,1444	193,1448	273,10158
**Buprofezin**	$C_{16}H_{23}N_3OS$	Positive	15,06	105,0573	175,0865	249,10571
**Butafenacil**	$C_{20}H_{18}ClF_3N_2O_6$	Positive	19,18	331,0092	179,9847	123,9949
**Butilate**	$C_{11}H_{23}NOS$	Positive	8,3	57,07042	146,064	156,13883
**Butralin**	$C_{14}H_{21}N_3O_4$	Positive	13,43	266,1136	250,1185	190,11006
**Cadusafos**	$C_{10}H_{23}O_2PS_2$	Positive	10,45	158,9697	130,9385	157,96194
**Carbophenothion**	$C_{11}H_{16}ClO_2PS_3$	Positive	16,17	156,9873	124,9821	295,98559
**Carbophenothion methyl**	$C_9H_{12}ClO_2PS_3$	Positive	15,63	156,9547	124,9826	142,97222
**Chinomethionate**	$C_{10}H_6N_2OS_2$	Positive	14,46	205,9966	233,9916	116,04946
**Chlordane (CIS+TRANS)**	$C_{10}H_6Cl_8$	Positive	14,43	372,8254	374,8224	236,8409
**Chlorfenapyr**	$C_{15}H_{11}BrClF_3N_2O$	Positive	15,24	247,0478	363,9403	137,00269
**Chlorfenprop methyl**	$C_{10}H_{10}Cl_2O_2$	Positive	9,66	125,0158	165,0107	196,0291
**Chlorfenson**	$C_{12}H_8Cl_2O_3S$	Positive	14,72	174,9614	128,107	110,9996
**Chlorfenvinphos**	$C_{12}H_{14}Cl_3O_4P$	Positive	13,85	266,9377	322,9999	294,96866
**Chlorflurenol-methyl**	$C_{15}H_{11}ClO_3$	Positive	14,21	215,0264	217,0232	151,05477
**Chlormephos**	$C_5H_{12}ClO_2PS_2$	Positive	8,39	121,0412	96,9507	154,02118
**Chloropropylate**	$C_{17}H_{16}Cl_2O_3$	Positive	15,54	138,9946	251,0026	252,99953
**Chlorothalonil**	$C_8Cl_4N_2$	Positive	11,53	265,8779	263,881	132,9713

**Chlorpropham**	$C_{10}H_{12}ClNO_2$	Positive	10,17	127,0183	171,0082	213,0551
**Chlorpyrifos – ethyl**	$C_9H_{11}Cl_3NO_3PS$	Positive	13,12	196,9196	257,8943	313,95688
**Chlorpyrifos – methyl**	$C_7H_7Cl_3NO_3PS$	Positive	12,29	285,9256	124,982	78,99433

Table S1. (Continued)

Compound name	Elemental Composition	Polarity	RT	Quan ion (*m*/*z*)	Confirming ion 1 (*m*/*z*)	Confirming ion 2 (*m*/*z*)
**Chlorthion**	$C_8H_9ClO_5PS$	Positive	13,37	108,9877	124,9826	127,9697
**Chlozolinate**	$C_{13}H_{11}Cl_2NO_5$	Positive	13,79	187,9841	331,0007	123,9949
**Cinidon-ethyl**	$C_{19}H_{17}Cl_2NO_4$	Positive	19,18	331,0092	179,9846	123,99494
**Clodinafop-propargyl**	$C_{17}H_{13}ClFNO_4$	Positive	16,26	238,0066	266,0379	349,05116
**Crimidine**	$C_7H_{10}ClN_3$	Positive	9,01	142,0292	156,0323	171,05566
**Cyanofenphos**	$C_{15}H_{14}NO_2PS$	Positive	16,17	156,9871	169,0412	141,00999
**Cyanophos**	$C_9H_{10}NO_3PS$	Positive	11,25	124,9826	227,9884	211,99351
**Cycloate**	$C_{11}H_{21}NOS$	Positive	10,06	154,1226	83,08553	186,09465
**Cyflufenamid**	$C_{20}H_{17}F_5N_2O_2$	Positive	15,14	223,0288	118,0413	412,12015
**Cyfluthrin**	$C_{22}H_{18}Cl_2FNO_3$	Positive	19,68	206,0601	199,0554	163,0076
**Cypermethrin**	$C_{22}H_{19}Cl_2NO_3$	Positive	19,8	181,0648	209,0838	152,06207
**Cyproconazole**	$C_{15}H_{18}ClN_3O$	Positive	15,37	222,0427	138,9945	125,01528
**Cyprodinil**	$C_{14}H_{15}N_3$	Positive	13,72	224,1188	210,1031	77,03912
**DCPA**	$C_{10}H_6Cl_4O_4$	Positive	13,26	300,8801	331,8985	272,88525
**Deltamethrin**	$C_{22}H_{19}Br_2NO_3$	Positive	21,47	181,0648	252,9046	171,98822
**Diazinone**	$C_{12}H_{21}N_2O_3PS$	Positive	11,33	137,071	179,1179	199,06309
**Dichlobenil**	$C_7H_3Cl_2N$	Positive	7,71	170,9636	164,0009	100,01815
**Dichlofenthion**	$C_{10}H_{13}Cl_2O_3PS$	Positive	12,13	222,9379	279,0006	161,96328
**Dichloran**	$C_6H_4Cl_2N_2O_2$	Positive	10,93	175,9664	123,9949	205,96445
**Dichlorvos**	$C_4H_7Cl_2O_4P$	Positive	6,88	127,0154	184,9765	109,00495
**Diclofop-methyl**	$C_{16}H_{14}Cl_2O_4$	Positive	16,59	252,9823	281,0136	120,05751
**Dicofol. 4.4**	$C_{14}H_9Cl_5O$	Positive	15,54	138,9945	251,0025	215,02636
**Dieldrin**	$C_{12}H_8Cl_6O$	Positive	15,17	262,8564	236,8407	276,91387
**Difenoconazole**	$C_{19}H_{17}Cl_2N_3O_3$	Positive	21,19	264,982	323,0236	202,01799
**Diflufenican**	$C_{19}H_{11}F_5N_2O_2$	Positive	16,61	266,0422	246,0362	218,04122
**Dimethomorph**	$C_{21}H_{22}ClNO_4$	Positive	21,79	301,0632	165,0547	303,05987
**Diniconazole**	$C_{15}H_{17}Cl_2N_3O$	Positive	15,68	268,0044	270,0011	232,02776
**Diphenylamine**	$C_{12}H_{11}N$	Positive	9,99	169,0885	167,0729	141,06986
**Disulfoton**	$C_8H_{19}O_2PS_3$	Positive	11,56	88,03467	153,0139	185,9938
**Ditalimfos**	$C_{12}H_{14}NO_4PS$	Positive	14,53	130,0288	242,975	148,03931
**Edifenphos**	$C_{14}H_{15}O_2PS_2$	Positive	16,23	109,0112	201,0139	110,01902
**Endosulfan alpha**	$C_9H_6Cl_6O_3S$	Positive	12,07	236,8407	169,9684	276,87193
**Endosulfan beta**	$C_9H_6Cl_6O_3S$	Positive	14,68	271,8096	236,8406	169,96834
**Endosulfan ether**	$C_9H_6Cl_6O$	Positive	12,13	276,8721	306,8827	271,9096
**Endosulfan sulfate**	$C_9H_6Cl_6O_4S$	Positive	16,41	271,8097	236,8407	228,8954
**Endrin**	$C_{12}H_8Cl_6O$	Positive	15,56	262,8564	242,953	280,9267
**Endrin ketone**	$C_{12}H_8Cl_6O$	Positive	17,29	316,9034	314,9069	344,8983
**EPTC (Eptam)**	$C_9H_{19}NOS$	Positive	7,75	128,107	132,0841	160,0791
**Esfenvalerate**	$C_{25}H_{22}ClNO_3$	Positive	20,73	125,0153	225,0785	167,06223
**Ethion**	$C_9H_{22}O_4P_2S_4$	Positive	15,63	230,9732	202,9418	124,98212
**Ethoprophos**	$C_8H_{19}O_2PS_2$	Positive	9,99	157,962	96,95076	126,9978
**Ethoxyquin**	$C_{14}H_{19}NO$	Positive	10,9	202,1225	174,0913	145,08859
**Etridiazole**	$C_5H_5Cl_3N_2OS$	Positive	8,53	182,9181	210,9494	139,9123
**Etrimfos**	$C_{10}H_{17}N_2O_4PS$	Positive	11,64	153,0658	181,0971	292,06416
**Famfur (Fonofos)**	$C_{10}H_{16}NO_5PS_2$	Positive	16,04	124,9826	217,0088	92,98844
**Fenamiphos**	$C_{13}H_{22}NO_3PS$	Positive	14,55	154,0446	303,1052	260,05047
**Fenamiphos sulfone**	$C_{13}H_{22}NO_5PS$	Positive	16,97	292,0404	320,0717	214,06284
**Fenamiphos sulfoxide**	$C_{13}H_{22}NO_4PS$	Positive	16,9	304,0767	196,0791	122,03655
**Fenarimol**	$C_{17}H_{12}Cl_2N_2O$	Positive	16,64	138,9946	107,024	219,03198

**Fenazaquin**	$C_{20}H_{22}N_2O$	Positive	17,6	145,0402	160,1252	117,07042
**Fenbuconazol**	$C_{19}H_{17}ClN_4$	Positive	19,53	129,0578	198,0905	125,0158
**Fenchlorphos/Ronnel**	$C_8H_8Cl_3O_3PS$	Positive	12,6	284,9303	124,9821	269,90686

Table S1. (Continued)

Compound name	Elemental Composition	Polarity	RT	Quan ion (*m*/*z*)	Confirming ion 1 (*m*/*z*)	Confirming ion 2 (*m*/*z*)
**Fenhexamid**	$C_{14}H_{17}Cl_2NO_2$	Positive	16,46	97,10172	176,9748	266,09478
**Fenitrothion**	$C_9H_{12}NO_5PS$	Positive	12,78	260,0142	124,9822	277,01703
**Fenobucarb**	$C_{12}H_{17}NO_2$	Positive	9,74	150,1045	103,0548	135,08099
**Fenoxaprop-P-ethyl**	$C_{18}H_{16}ClNO_5$	Positive	18,68	288,0427	261,0193	361,0717
**Fenoxycarb**	$C_{17}H_{19}NO_4$	Positive	17,21	88,0393	116,0706	186,0675
**Fenpropathrin**	$C_{22}H_{23}NO_3$	Positive	17,35	181,0648	265,0734	209,0836
**Fenpropimorph**	$C_{20}H_{33}NO$	Positive	13,19	128,1075	173,1075	117,07042
**Fenson**	$C_{12}H_9ClO_3S$	Positive	13,47	77,03861	141,0004	267,99573
**Fenthion**	$C_{10}H_{15}O_3PS_2$	Positive	13,13	278,0194	169,0139	124,98216
**Fenvalerate**	$C_{25}H_{22}ClNO_3$	Positive	20,92	225,0785	125,0153	167,06223
**Fipronil**	$C_{12}H_4Cl_2F_6N_4OS$	Positive	13,77	366,9426	212,9479	254,96975
**Fipronil sulfone**	$C_{12}H_4Cl_2F_6N_4O_2S$	Positive	14,85	254,9699	212,948	177,97913
**Fluchloralin**	$C_{12}H_{13}ClF_3N_3O_4$	Positive	11,4	264,0227	306,0696	326,01499
**Flucythrinate**	$C_{26}H_{23}F_2NO_4$	Positive	20,15	157,046	199,0929	181,06477
**Fludioxonil**	$C_{12}H_6F_2N_2O_2$	Positive	14,74	248,0391	127,0417	154,05247
**Flumetralin**	$C_{16}H_{12}ClF_4N_3O_4$	Positive	14,43	143,0058	156,9851	404,04211
**Flumioxazin**	$C_{19}H_{15}FN_2O_4$	Positive	20,69	354,1009	259,0514	287,08267
**Fluotrimazole**	$C_{22}H_{16}F_3N_3$	Positive	16,69	165,0699	311,1042	233,05726
**Fluquinconazole**	$C_{16}H_8Cl_2FN_5O$	Positive	19,21	340,0401	313,0292	286,01834
**Fluvalinate (Tau)**	$C_{26}H_{22}ClF_3N_2O_3$	Positive	20,84	250,0606	181,0648	205,99797
**Fonofos**	$C_{10}H_{15}OPS_2$	Positive	11,39	108,9872	137,0185	246,02965
**Formothion**	$C_6H_{12}NO_4PS_2$	Positive	11,9	93,01003	142,9926	110,96638
**Furalaxyl**	$C_{17}H_{19}NO_4$	Positive	13,98	95,0127	242,1175	152,07054
**Halfenprox**	$C_{24}H_{23}BrF_2O_3$	Positive	19,89	262,9883	183,051	264,9858
**Heptachlor**	$C_{10}H_5Cl_7$	Positive	12,65	271,8098	236,8407	100,00747
**Heptachlor-epoxide-A-endo (cis)**	$C_{10}H_5Cl_7O$	Positive	13,99	352,8434	262,8565	236,84085
**Heptachlor-epoxide-A-endo (trans)**	$C_{10}H_5Cl_7O$	Positive	14,07	236,8407	252,8953	352,84351
**Heptenophos**	$C_9H_{12}ClO_4P$	Positive	9,44	89,03857	124,0075	215,04675
**Hexachlorocychlohexane-Alpha**	$C_6H_6Cl_6$	Positive	10,73	180,9372	182,9342	218,9109
**Hexachlorocyclohexane-Beta**	$C_6H_6Cl_6$	Positive	11,2	180,9372	182,9342	218,91092
**Hexachlorocyclohexane-Delta**	$C_6H_6Cl_7$	Positive	11,75	180,9372	182,9342	218,91096
**Hexaconazole**	$C_{14}H_{17}Cl_2N_3O$	Positive	14,77	82,04052	231,0343	256,00444
**Hexazinone**	$C_{12}H_{20}N_4O_2$	Positive	16,45	171,0877	83,02402	128,08189
**Indoxacarb**	$C_{22}H_{17}ClF_3N_3O_7$	Positive	21,26	203,0194	218,0429	235,04562
**Iodofenphos**	$C_8H_8Cl_2IO_3PS$	Positive	14,77	376,8658	124,9821	258,85727
**Iprodione**	$C_{13}H_{13}Cl_2N_3O_3$	Positive	17,05	314,0095	244,9881	316,00662
**Isobenzan**	$C_9H_4Cl_8O$	Positive	13,54	102,9951	310,8331	374,8047
**Isocarbophos**	$C_{11}H_{16}NO_4PS$	Positive	13,27	135,9976	121,0283	230,0033
**Isodrin**	$C_{12}H_8Cl_6$	Positive	13,85	192,9373	262,8564	146,9762
**Isofenphos**	$C_{15}H_{24}NO_4PS$	Positive	13,81	184,9997	213,0309	121,02839
**Isofenphos-methyl**	$C_{14}H_{22}NO_4PS$	Positive	13,56	199,0154	121,0285	230,98742
**Isomethiozin**	$C_{12}H_{20}N_4OS$	Positive	13,17	225,081	198,0701	184,05445
**Isopropalin**	$C_{15}H_{23}N_3O_4$	Positive	13,54	280,1291	238,0821	264,13438
**Isoprothiolane**	$C_{12}H_{18}O_4S_2$	Positive	14,7	117,9906	161,9804	188,96744

**Kresoxim-methyl**	$C_{18}H_{19}NO_4$	Positive	14,99	116,0495	131,0729	206,08101
**Lambda Cyhalothrin**	$C_{23}H_{19}ClF_3NO_3$	Positive	18,13	181,0648	141,0511	197,03389

Table S1. (Continued)

Compound name	Elemental Composition	Polarity	RT	Quan ion (*m/z*)	Confirming ion 1 (*m/z*)	Confirming ion 2 (*m/z*)
**Lenacile**	$C_{13}H_{28}N_2O_3$	Positive	15,54	153,0664	110,0606	154,06871
**Leptophos**	$C_{13}H_{10}BrCl_2O_2PS$	Positive	17,98	171,0027	376,8982	155,02564
**Lindane-gamma**	$C_6H_6Cl_6$	Positive	11,3	180,9371	182,9342	218,9109
**Malathion**	$C_{10}H_{19}O_6PS_2$	Positive	12,87	127,039	173,0808	142,99263
**Mefenpyr-diethyl**	$C_{16}H_{18}Cl_2N_2O_4$	Positive	16,84	299,0354	252,9935	327,03032
**Mepanipyrim**	$C_{14}H_{13}N_3$	Positive	14,49	222,1031	207,0796	77,03912
**Metalaxyl**	$C_{15}H_{21}NO_4$	Positive	12,48	160,1119	206,1174	132,08076
**Metazachlor**	$C_{14}H_{16}ClN_3O$	Positive	13,75	81,04527	133,0891	132,08132
**Methamidophos**	$C_2H_8NO_2PS$	Positive	6,76	95,01307	141,0008	125,9774
**Methidation**	$C_6H_{11}N_2O_4PS_3$	Positive	14,25	145,0066	85,03964	124,98207
**Methoxychlor**	$C_{16}H_{15}Cl_3O_2$	Positive	16,59	227,1066	169,0522	212,08322
**Metolachlor**	$C_{15}H_{22}ClNO_2$	Positive	13,1	238,0999	240,0967	211,07639
**Mevinphos**	$C_7H_{13}O_6P$	Positive	8,2	127,0155	164,0233	192,01819
**Mirex**	$C_{10}Cl_{12}$	Positive	18,49	271,8097	236,8408	269,81257
**Myclobutanil**	$C_{15}H_{17}ClN_4$	Positive	14,98	179,0244	150,0105	245,05894
**Napropamide**	$C_{17}H_{21}NO_2$	Positive	14,68	72,08132	128,1075	100,07623
**Nicotine**	$C_{10}H_{14}N_2$	Positive	7,81	84,08132	133,0766	161,10787
**Nitrofen**	$C_{12}H_7Cl_2NO_3$	Positive	15,4	202,018	282,9799	139,05421
**Nitrothal isopropyl**	$C_{14}H_{17}NO_6$	Positive	13,32	194,0084	212,019	236,05544
**Norflurazon**	$C_{12}H_9ClF_3N_3O$	Positive	16,19	173,0321	145,026	303,03827
**Nuarimol**	$C_{17}H_{12}ClFN_2O$	Positive	16,64	138,9946	235,0321	203,0614
**o.p'-DDD (Mitotane)**	$C_{14}H_{10}Cl_4$	Positive	15,74	235,0076	165,0699	176,06189
**o.p'-DDE (2.4-DDE)**	$C_{14}H_8Cl$	Positive	14,41	245,9999	247,9968	176,06203
**o.p'-DDT**	$C_{14}H_9Cl_5$	Positive	15,82	235,0077	199,0309	237,00468
**Oxadiazone**	$C_{15}H_{18}Cl_2N_2O_3$	Positive	14,88	174,9586	258,0321	302,02194
**Oxadixyl**	$C_{14}H_{18}N_2O_4$	Positive	15,65	132,0808	163,0991	233,09204
**Oxyfluorfen**	$C_{15}H_{11}ClF_3NO_4$	Positive	14,96	252,039	300,0031	317,00583
**p.p´-DDD**	$C_{14}H_{10}Cl_4$	Positive	15,12	235,0076	165,0699	199,03076
**p.p´-DDE**	$C_{14}H_8Cl_4$	Positive	14,98	245,9999	176,062	315,93738
**p.p´-DDT**	$C_{14}H_9Cl_5$	Positive	16,44	235,0076	165,0699	199,03090
**Parathion**	$C_{10}H_{14}NO_5PS$	Positive	13,2	96,95077	109,005	291,03259
**Parathion-methyl**	$C_8H_{10}NO_5PS$	Positive	12,34	124,982	263,0014	109,00501
**Penconazole**	$C_{13}H_{15}Cl_2N_3$	Positive	13,82	158,9763	248,0949	160,97333
**Pendimethalin**	$C_{13}H_{19}N_3O_4$	Positive	13,73	252,0979	162,0788	191,0689
**Pentachloroaniline**	$C_6H_2Cl_5N$	Positive	12,17	264,8594	262,8624	191,9169
**Pentachloroanisole**	$C_7H_3Cl_5O$	Positive	10,96	236,8407	264,8359	279,85928
**Pentachlorobenzene**	C_6HCl_5	Positive	9,24	214,8798	212,8832	177,91438
**Permethrine**	$C_{21}H_{20}Cl_2O_3$	Positive	19,07	183,0804	163,0076	127,03097
**Perthane**	$C_{18}H_{20}Cl_2$	Positive	15,36	223,1479	167,0854	178,07753
**Phenisopham**	$C_{19}H_{22}N_2O_4$	Positive	18,94	148,0762	120,0813	282,10044
**Phenol 2.4.6-trichloro**	$C_6H_2Cl_3OH$	Positive	7,83	195,9242	197,9214	96,98392
**Phenthoate**	$C_{12}H_{17}O_4PS_2$	Positive	13,93	121,0106	273,9883	245,99332
**Phosalone**	$C_{12}H_{15}ClNO_4PS_2$	Positive	17,9	182,0003	121,0414	96,9508
**Phosmet**	$C_{11}H_{12}NO_4PS_2$	Positive	17,22	160,0393	133,0285	161,04253
**Phthalimide**	$C_8H_5NO_2$	Positive	8,63	147,0314	103,0416	76,03072
**Piperonylbutoxide**	$C_{19}H_{30}O_5$	Positive	16,67	176,0837	193,0865	177,09155
**Piperophos**	$C_{14}H_{28}NO_3PS_2$	Positive	17,26	140,1075	122,097	97,08914
**Pirimicarb**	$C_{11}H_{18}N_4O_2$	Positive	11,78	166,098	72,04493	238,14297

**Pirimiphos-ethyl**	$C_{13}H_{24}N_3O_3PS$	Positive	13,42	168,0588	180,113	318,10342
**Pirimiphos-methyl**	$C_{11}H_{20}N_3O_3PS$	Positive	12,71	290,0723	276,0566	180,11299
**Plifenat**	$C_{10}H_7Cl_5O_2$	Positive	12,32	216,9823	241,9032	257,8982

Table S1. (Continued)

Compound name	Elemental Composition	Polarity	RT	Quan ion (*m*/*z*)	Confirming ion 1 (*m*/*z*)	Confirming ion 2 (*m*/*z*)
**Prallethrin**	$C_{19}H_{24}O_3$	Positive	14,11	123,1174	81,07042	77,03912
**Prochloraz**	$C_{15}H_{16}Cl_3N_3O_2$	Positive	19,26	180,1131	308,0005	70,02876
**Procymidone**	$C_{13}H_{11}Cl_2NO_2$	Positive	14,09	96,05694	283,0161	255,02155
**Profenofos**	$C_{11}H_{15}BrClO_3PS$	Positive	14,83	207,9105	336,9657	266,88759
**Profluralin**	$C_{14}H_{16}F_3N_3O_4$	Positive	11,2	318,0693	330,1059	186,03982
**Prometryn**	$C_{10}H_{19}N_5S$	Positive	12,48	184,0657	226,1126	199,08916
**Propachlor**	$C_{11}H_{14}ClNO$	Positive	9,82	120,0808	176,107	93,05731
**Propanil**	$C_9H_9Cl_2NO$	Positive	12,16	160,9792	162,9763	217,00549
**Propargite**	$C_{19}H_{26}O_4S$	Positive	16,61	135,0805	173,0961	107,04921
**Propham**	$C_{10}H_{13}NO_2$	Positive	8,51	93,05729	137,0471	179,09398
**Propiconazole**	$C_{15}H_{17}Cl_2N_3O_2$	Positive	16,36	172,9556	259,029	190,96602
**Propoxur**	$C_{11}H_{15}NO_3$	Positive	9,75	110,0361	152,0831	81,0335
**Propyzamide**	$C_{12}H_{11}Cl_2NO$	Positive	11,35	172,9561	144,9612	239,99829
**Prothiofos**	$C_{11}H_{15}Cl_2O_2PS_2$	Positive	14,79	112,928	161,9634	308,99332
**Pyrazophos**	$C_{14}H_{20}N_3O_5PS$	Positive	18,37	221,0795	232,1081	265,08807
**Pyridaben**	$C_{19}H_{25}ClN_2OS$	Positive	19,14	147,1168	117,0699	309,08243
**Pyridalyl**	$C_{18}H_{14}Cl_4F_3NO_3$	Positive	20,16	204,0631	164,0318	148,03686
**Pyridaphenthion**	$C_{14}H_{17}N_2O_4PS$	Positive	17,03	199,0865	96,95085	340,06412
**Pyrifenox**	$C_{14}H_{12}Cl_2N_2O$	Positive	14,35	262,006	186,9586	170,96371
**Pyrimethanil**	$C_{14}H_{12}Cl_2N_2O$	Positive	11,48	198,1024	183,0791	118,05251
**Pyriproxyfen**	$C_{20}H_{19}NO_3$	Positive	17,98	136,0757	96,0444	226,09888
**Quinalphos**	$C_{12}H_{15}N_2O_3PS$	Positive	13,98	146,0475	157,076	118,05255
**Quinoxyfen**	$C_{15}HCl_2FNO$	Positive	16,33	237,059	272,0278	112,03244
**Quintozene**	$C_6Cl_5NO_2$	Positive	10,96	236,8408	213,8719	141,93721
**Resmethrin**	$C_{22}H_{26}O_3$	Positive	16,7	123,1174	171,081	81,07042
**S421 (Octachlorodipropyl ether)**	$C_6H_6Cl_8O$	Positive	12,73	129,9139	180,113	82,94501
**Silafluofen**	$C_{25}H_{29}FO_2Si$	Positive	20,27	179,0889	286,1185	276,09804
**Simazina**	$C_7H_{12}ClN_5$	Positive	10,91	186,0546	173,0468	158,02334
**Spiromesifen**	$C_{23}H_{30}O_4$	Positive	16,95	272,1412	99,08099	254,13068
**Spiroxamine**	$C_{18}H_{35}NO_2$	Positive	12,36	100,1126	126,1283	198,1494
**Sulfotep**	$C_8H_{20}O_5P_2S_2$	Positive	10,29	322,0223	237,9283	201,98817
**Sulprofos**	$C_{12}H_{19}O_2PS_3$	Positive	15,95	156,0062	140,0291	112,92794
**Tebuconazole**	$C_{16}H_{22}ClN_3O$	Positive	16,64	125,0153	250,0742	163,0309
**Tebufenpyrad**	$C_{18}H_{24}ClN_3O$	Positive	17,46	171,0325	318,1373	276,09036
**Tecnazene**	$C_6HCl_4NO_2$	Positive	9,81	202,8796	107,9761	177,91367
**Tefluthrine**	$C_{17}H_{14}ClF_7O_2$	Positive	11,54	177,0322	141,051	197,03384
**Terbacil**	$C_9H_{13}ClN_2O_2$	Positive	11,55	161,0118	116,9981	75,97159
**Terbufos**	$C_9H_{21}O_2PS_3$	Positive	11,26	57,07042	230,9737	103,05814
**Terbumeton**	$C_{10}H_{19}N_5O$	Positive	11,07	210,1355	169,0964	154,07288
**Terbuthylazine**	$C_9H_{16}ClN_5$	Positive	11,24	214,0859	173,0468	216,08257
**Terbutryn**	$C_{10}H_{19}N_5S$	Positive	12,74	170,0495	185,0729	226,11209
**Tetrachlorvinphos**	$C_{10}H_9Cl_4O_4P$	Positive	14,37	330,9267	127,0155	328,92925
**Tetraconazole**	$C_{13}H_{11}Cl_2F_4N_3O$	Positive	13,23	336,0517	170,9761	158,97623
**Tetradiphon**	$C_{12}H_6Cl_4O_2S$	Positive	17,82	158,9666	226,8887	139,00585
**Tetramethrin**	$C_{19}H_{25}NO_4$	Positive	17,18	164,0712	123,1174	79,05477
**Tetrasul**	$C_{12}H_6Cl_4S$	Positive	16,02	251,9562	323,8908	175,9249

**Thiobencarb**	$C_{12}H_{16}ClNOS$	Positive	13,07	100,0757	125,0153	257,0636
**Thiofanox**	$C_9H_{18}N_2O_2S$	Positive	7,2	161,0874	61,01119	83,07349
**Thiometon**	$C_6H_{15}O_2PS_3$	Positive	10,75	88,03467	124,9826	60,00337

Table S1. (Continued)

Compound name	Elemental Composition	Polarity	RT	Quan ion (*m*/*z*)	Confirming ion 1 (*m*/*z*)	Confirming ion 2 (*m*/*z*)
**Tolclofos-methyl**	$C_9H_{11}Cl_2O_3PS$	Positive	12,39	264,9849	124,9821	249,9616
**Tolylfluanid**	$C_{10}H_{13}Cl_2FN_2O_2S_2$	Positive	13,86	137,0299	237,966	239,8997
**Transfluthrin**	$C_{15}H_{12}Cl_2F_4O_2$	Positive	12,33	163,0164	127,0309	143,01026
**Triadimefon**	$C_{14}H_{16}ClN_3O_2$	Positive	13,27	208,0278	85,06534	128,00289
**Triadimenol**	$C_{14}H_{18}ClN_3O_2$	Positive	14,19	168,1137	128,0029	208,02776
**Triazophos**	$C_{12}H_{16}N_3O_3PS$	Positive	15,87	161,0589	91,04219	285,03369
**Trichloronat**	$C_{10}H_{12}Cl_3O_2PS$	Positive	13,47	108,9872	268,9354	296,96655
**Trifloxystrobin**	$C_{20}H_{19}F_3N_2O_4$	Positive	16,07	172,0374	145,0265	222,07663
**Trifluralin**	$C_{13}H_{16}F_3N_3O_4$	Positive	10,23	264,0226	306,0695	248,02771
**Vinclozoline**	$C_{12}H_9Cl_2NO_3$	Positive	12,31	178,0418	212,0028	197,98714
**Zoxamida**	$C_{14}H_{16}Cl_3NO_2$	Positive	16,91	258,0452	158,9768	242,01394

Table S2. Summary of data obtained for validation tests performed to matrices studies

Compound	APPLE							APPLE JUICE						
	Linearity (R^2)	Recovery		Precision				Linearity (R^2)	Recovery		Precision			
		R (%)		Intraday (%)		Interday (%)			R (%)		Intraday (%)		Interday (%)	
		1 µg kg^{-1}	10 µg kg^{-1}	1 µg kg^{-1}	10 µg kg^{-1}	1 µg kg^{-1}	10 µg kg^{-1}		1 µg L^{-1}	10 µg L^{-1}	1 µg L^{-1}	10 µg L^{-1}	1 µg L^{-1}	10 µg L^{-1}
**1,4-dimethylnaphthalene**	0.99	87	90	2	1	3	8	0.99	95	70	2	9	3	17
**2-phenylphenol**	0.99	90	93	2	1	20	19	0.99	95	77	3	9	7	19
**3,5-Dichloroaniline**	0.99	79	85	1	3	20	16	0.99	110	91	5	5	9	15
**4,4Dibromobenzophenone**	0.99	91	94	3	2	18	20	0.98	120	70	8	9	14	11
**4,4-Dichlorobenzophenone**	0.99	88	90	1	3	13	2	0.98	119	78	3	10	7	15
**4-Chloro-3-methylphenol**	0.99	103	97	1	2	5	14	0.98	103	96	9	14	3	18
**Aclonifen**	0.99	90	97	19	2	18	20	0.98	114	71	9	14	16	14
**Acrinathrin**	0.99	96	96	20	1	14	19	0.99	<50	<50	>23	>23	>23	16
**Alachlor**	0.99	76	79	2	1	11	4	0.99	115	80	5	10	4	18
**Aldrin**	0.99	79	80	2	1	4	5	0.99	118	89	1	10	2	13
**Anthraquinone**	0.99	87	89	1	1	18	17	0.98	120	113	9	8	10	11
**Atrazine**	0.99	90	92	2	1	11	4	0.98	115	81	4	11	3	18
**Azoxystrobin**	0.99	103	90	20	2	*	20	0.99	>120	70	20	13	20	15
**Benalaxyl**	0.99	99	94	7	1	12	5	0.98	120	74	7	11	3	12
**Benfluralin**	0.99	78	79	3	1	8	8	0.98	119	72	2	11	8	18
**Benfuresate**	0.99	73	79	3	1	12	2	0.99	117	87	2	9	3	17
**Benodalil**	0.99	98	96	4	3	20	8	0.98	120	73	6	12	5	18
**Benoxacor**	0.99	103	100	2	1	4	8	0.99	120	75	4	9	13	13
**Benzene, hexachloro**	0.99	78	79	1	1	13	4	0.99	109	70	3	8	1	11
**Benzyl benzoate**	0.99	86	96	11	1	19	19	<0.98	109	70	6	22	15	16
**Bifenazato**	0.98	88	97	*	18	18	20	0.99	116	75	9	12	20	16
**Bifenox**	0.99	88	90	*	14	*	18	0.99	<70	71	4	12	*	12
**Bifenthrin**	0.99	94	98	4	1	2	7	0.98	120	72	8	11	11	14
**Biphenyl**	0.99	98	96	2	1	18	7	0.99	106	73	1	8	1	16
**Bitertanol**	0.99	103	97	13	1	20	15	0.98	>120	73	5	14	6	11
**Boscalid**	0.99	89	90	1	2	20	5	0.98	120	71	10	10	7	14
**Bromacil**	0.99	97	101	5	1	3	6	0.98	120	73	6	9	6	15
**Bromocyclen**	0.99	93	87	1	1	6	5	0.99	119	71	8	10	11	14
**Bromophos-ethyl**	0.99	90	98	4	1	20	5	0.98	118	73	4	11	5	18
**Bromophos-methyl**	0.99	101	89	3	1	8	7	0.98	120	73	4	9	12	18
**Bromopropylate**	0.99	87	95	4	1	12	6	0.99	120	72	8	12	19	20
**Bupirimate**	0.99	90	91	2	1	7	5	0.98	119	74	6	10	7	15
**Buprofezin**	0.99	79	89	4	1	19	9	0.98	106	79	4	10	8	11
**Butafenacil**	0.99	102	97	7	1	18	15	0.98	>120	72	20	13	20	12
**Butilate**	0.99	108	87	10	1	11	3	0.99	109	76	9	8	3	11
**Butralin**	0.99	98	89	5	1	1	5	0.98	119	71	9	10	8	19
**Cadusafos**	0.99	87	94	3	2	2	2	0.98	120	78	5	8	5	12

*acceptable value at 5 ppb

Table S2. (Continued)

Compound	APPLE							APPLE JUICE						
	Linearity (R^2)	Recovery		Precision				Linearity (R^2)	Recovery		Precision			
		R (%)		Intraday (%)		Interday (%)			R (%)		Intraday (%)		Interday (%)	
		1 µg kg^{-1}	10 µg kg^{-1}	1 µg kg^{-1}	10 µg kg^{-1}	1 µg kg^{-1}	10 µg kg^{-1}		1 µg L^{-1}	10 µg L^{-1}	1 µg L^{-1}	10 µg L^{-1}	1 µg L^{-1}	10 µg L^{-1}
**Carbophenothion**	0.99	90	98	3	1	11	9	0.99	112	73	12	12	19	15
**Carbophenothion methyl**	0.98	90	97	*	7	*	18	0.99	120	70	20	15	17	13
**Chinomethionate**	0.99	79	93	9	3	20	16	0.98	70	72	11	9	17	12
**Chlordane (CIS+TRANS)**	0.99	96	92	20	2	16	10	0.99	119	74	4	10	15	11
**Chlorfenapyr**	0.99	98	87	8	2	6	4	0.99	107	76	13	10	*	14
**Chlorfenprop methyl**	0.99	68	78	4	2	16	9	0.99	111	77	1	9	10	15
**Chlorfenson**	0.99	88	90	2	1	14	8	0.99	110	81	2	10	8	18
**Chlorfenvinphos**	0.99	79	94	4	1	4	1	0.98	>120	73	8	11	10	19
**Chlorflurenol-methyl**	0.99	89	92	3	1	9	6	0.98	120	85	6	10	5	19
**Chlormephos**	0.99	70	83	3	1	10	8	0.99	107	74	4	9	7	20
**Chloropropylate**	0.99	109	101	2	1	7	4	0.99	97	80	6	10	3	20
**Chlorothalonil**	0.99	103	87	1	1	5	13	0.98	<70	79	*	13	4	20
**Chlorpropham**	0.99	89	88	3	1	20	18	0.99	118	70	7	9	5	11
**Chlorpyrifos – ethyl**	0.99	96	97	7	1	19	7	0.99	117	70	3	9	7	12
**Chlorpyrifos – methyl**	0.99	90	94	1	1	10	6	0.99	120	71	4	9	6	18
**Chlorthion**	0.98	88	98	5	1	*	20	0.99	98	72	4	11	19	12
**Chlozolinate**	0.99	90	94	7	1	8	2	0.99	111	74	10	9	13	12
**Cinidon-ethyl**	0.99	76	79	2	4	19	15	0.98	>120	<70	15	13	19	15
**Clodinafop-propargyl**	0.99	97	89	10	1	20	19	<0.98	117	74	10	8	15	16
**Crimidine**	0.99	66	75	1	1	20	18	0.99	104	74	1	9	4	12
**Cyanofenphos**	0.99	87	102	3	1	11	9	0.98	119	79	9	10	3	14
**Cyanophos**	0.99	97	106	7	1	13	11	0.99	120	98	6	12	3	20
**Cycloate**	0.99	79	86	2	1	9	4	0.99	116	79	3	9	5	19
**Cyflufenamid**	0.99	90	95	*	2	19	20	0.98	<70	78	14	3	20	13
**Cyfluthrin**	0.99	104	98	*	4	15	15	<0.98	120	72	14	10	20	15
**Cypermethrin**	0.99	96	93	*	4	5	6	0.98	>120	86	9	20	10	19
**Cyproconazole**	0.99	79	89	4	1	13	9	0.98	116	77	13	11	10	19
**Cyprodinil**	0.99	89	103	4	1	23	5	0.98	120	73	6	8	12	15
**DCPA**	0.99	88	93	2	1	5	4	0.98	118	86	1	9	5	15
**Deltamethrin**	0.99	79	78	8	3	20	19	0.99	70	70	6	13	20	19
**Diazinone**	0.99	96	89	5	1	10	7	0.99	105	76	11	10	7	15
**Dichlobenil**	0.99	81	85	1	1	8	2	0.99	108	79	1	8	5	12
**Dichlofenthion**	0.99	76	86	2	1	5	6	0.99	118	86	4	10	5	12
**Dichloran**	0.99	87	83	7	2	20	18	0.99	120	72	8	10	15	13
**Dichlorvos**	0.99	85	93	3	1	5	5	0.99	>120	98	8	11	6	16
**Diclofop-methyl**	0.99	80	86	*	1	11	14	1.00	98	84	3	10	20	11
**Dicofol, 4,4**	0.99	79	98	2	1	7	4	0.99	97	73	6	10	3	18

*acceptable value at 5 ppb

Table S2. (Continued)

Compound	APPLE							APPLE JUICE						
	Linearity (R^2)	Recovery		Precision				Linearity (R^2)	Recovery		Precision			
		R (%)		Intraday (%)		Interday (%)			R (%)		Intraday (%)		Interday (%)	
		1 µg kg^{-1}	10 µg kg^{-1}	1 µg kg^{-1}	10 µg kg^{-1}	1 µg kg^{-1}	10 µg kg^{-1}		1 µg L^{-1}	10 µg L^{-1}	1 µg L^{-1}	10 µg L^{-1}	1 µg L^{-1}	10 µg L^{-1}
**Dieldrin**	0.99	106	98	3	2	18	7	0.99	120	>120	5	6	3	12
**Difenoconazole**	0.99	98	89	13	7	20	19	1.00	<70	84	*	16	20	20
**Diflufenican**	0.99	86	96	5	1	5	7	0.98	120	71	7	11	6	18
**Dimethomorph**	0.99	85	91	>23	>23	>23	>23	0.99	<70	70	*	16	20	18
**Diniconazole**	0.99	96	99	10	1	3	5	0.98	120	72	3	12	10	19
**Diphenylamine**	0.99	78	89	4	1	18	12	0.99	120	95	*	11	5	13
**Disulfoton**	0.99	94	94	5	1	20	5	0.99	<70	>120	*	13	*	18
**Ditalimfos**	0.99	89	97	1	2	15	11	0.98	87	49	4	10	6	14
**Edifenphos**	0.99	80	88	9	2	19	16	0.98	119	71	3	10	6	13
**Endosulfan alpha**	0.99	<50	<50	1	1	7	4	0.99	118	71	8	10	3	16
**Endosulfan beta**	0.99	<50	<50	20	9	12	20	0.99	104	79	0	10	7	16
**Endosulfan ether**	0.99	95	105	16	1	8	7	0.98	115	81	12	8	14	11
**Endosulfan sulfate**	0.99	93	89	1	6	18	11	0.99	117	78	8	9	16	15
**Endrin**	0.99	109	101	3	2	20	7	0.99	99	92	3	9	4	12
**Endrin ketone**	0.99	105	97	20	10	*	20	0.99	85	80	10	9	15	15
**EPTC (Eptam)**	0.99	98	103	7	3	2	7	0.99	118	79	5	9	1	13
**Esfenvalerate**	0.99	91	93	6	2	19	17	0.99	<70	49	*	15	7	18
**Ethion**	0.99	100	96	5	1	20	18	0.98	120	70	11	9	10	18
**Ethoprophos**	0.99	107	98	6	3	2	4	0.99	118	80	4	8	1	19
**Ethoxyquin**	0.99	78	85	11	20	18	20	0.99	<70	71	*	14	20	19
**Etridiazole**	0.99	83	91	6	1	12	5	0.99	108	72	7	4	14	18
**Etrimfos**	0.99	79	85	8	1	4	5	0.99	119	77	4	9	9	15
**Famfur (Fonofos)**	0.99	90	99	4	1	15	20	0.99	112	73	12	12	12	11
**Fenamiphos**	0.99	80	84	*	5	19	10	0.98	120	82	11	10	10	18
**Fenamiphos sulfone**	0.99	89	97	8	2	*	20	0.99	120	70	59	19	16	20
**Fenamiphos sulfoxide**	0.99	70	79	13	20	*	20	1.00	<70	<70	*	20	20	20
**Fenarimol**	0.99	79	84	6	3	20	7	0.99	118	70	4	11	20	16
**Fenazaquin**	0.99	84	89	*	20	*	9	0.99	115	<70	*	20	20	16
**Fenbuconazol**	0.99	87	100	6	2	9	11	0.99	>120	71	28	16	14	17
**Fenchlorphos/Ronnel**	0.99	79	87	1	1	8	6	0.99	117	75	6	11	3	16
**Fenhexamid**	0.99	103	97	3	1	3	19	0.99	115	71	2	6	20	19
**Fenitrothion**	0.99	77	87	15	1	4	17	0.99	120	75	6	11	3	19
**Fenobucarb**	0.99	86	98	9	2	3	17	0.99	95	82	4	7	15	16
**Fenoxaprop-P-ethyl**	0.99	79	87	8	1	20	8	0.99	100	70	20	11	20	19
**Fenoxycarb**	0.99	103	98	6	1	19	20	0.98	>120	71	14	13	15	14
**Fenpropathrin**	0.99	107	95	2	3	8	1	0.99	120	71	9	13	14	14
**Fenpropimorph**	0.99	95	103	4	1	9	6	0.99	120	86	4	9	7	10
**Fenson**	0.99	87	88	1	3	8	6	0.99	107	77	8	9	20	12

*acceptable value at 5 ppb

Table S2. (Continued)

Compound	APPLE							APPLE JUICE						
	Linearity (R^2)	Recovery		Precision				Linearity (R^2)	Recovery		Precision			
		R (%)		Intraday (%)		Interday (%)			R (%)		Intraday (%)		Interday (%)	
		1 µg kg^{-1}	10 µg kg^{-1}	1 µg kg^{-1}	10 µg kg^{-1}	1 µg kg^{-1}	10 µg kg^{-1}		1 µg L^{-1}	10 µg L^{-1}	1 µg L^{-1}	10 µg L^{-1}	1 µg L^{-1}	10 µg L^{-1}
**Fenthion**	0.99	81	90	3	2	8	7	0.98	120	80	6	10	2	15
**Fenvalerate**	0.99	95	102	*	2	173	173	0.99	<70	<70	*	15	20	12
**Fipronil**	0.99	97	110	7	2	*	7	0.99	119	74	7	9	6	13
**Fipronil sulfone**	0.99	104	89	9	2	17	6	0.99	80	76	12	11	18	16
**Fluchloralin**	0.99	67	81	6	1	2	20	<0.98	111	75	1	11	20	18
**Flucythrinate**	0.99	101	95	3	7	7	8	0.98	98	71	18	18	9	10
**Fludioxonil**	0.99	78	90	3	1	14	14	0.98	120	74	8	12	12	14
**Flumetralin**	0.99	84	93	10	1	12	6	0.99	111	74	1	8	5	16
**Flumioxazin**	0.99	93	96	7	5	*	17	0.99	<70	70	17	19	20	18
**Fluotrimazole**	0.99	78	79	2	2	16	6	1.00	89	77	8	11	20	13
**Fluquinconazole**	0.99	84	93	14	2	9	8	0.98	<70	71	20	10	20	15
**Fluvalinate (Tau)**	0.99	81	87	20	1	15	20	0.98	120	72	13	1	12	19
**Fonofos**	0.99	90	89	5	2	13	12	0.99	117	79	4	9	2	18
**Formothion**	0.99	92	100	8	1	20	19	0.99	98	70	3	9	10	18
**Furalaxyl**	0.99	106	98	8	2	8	3	0.99	118	77	3	9	12	19
**Halfenprox**	0.99	94	90	11	1	20	19	0.99	<70	70	5	8	19	16
**Heptachlor**	0.99	83	89	1	1	6	5	<0.98	120	73	12	8	18	14
**Heptachlor-epoxide-A-endo (cis)**	0.99	92	87	6	5	6	1	0.98	142	79	7	8	4	13
**Heptachlor-epoxide-A-endo (trans)**	0.99	94	97	7	2	6	19	0.99	115	78	5	8	9	16
**Heptenophos**	0.99	79	86	*	1	2	13	0.99	108	81	12	9	20	16
**Hexachlorocychlohexane-Alpha**	0.99	75	84	1	1	13	3	0.99	108	81	4	8	10	16
**Hexachlorocyclohexano-Beta**	0.99	<50	<50	5	3	6	3	0.98	116	82	1	10	4	12
**Hexachlorocyclohexano-Delta**	0.99	<50	<50	2	1	1	10	0.99	116	80	1	10	2	10
**Hexaconazole**	0.99	56	80	*	3	4	13	1.00	70	76	*	11	20	11
**Hexazinone**	0.99	88	98	5	1	7	9	0.98	90	75	4	12	14	17
**Indoxacarb**	0.99	78	79	*	3	*	19	1.00	70	92	*	10	20	15
**Iodofenphos**	0.99	106	89	2	1	12	13	0.98	98	70	12	10	13	20
**Iprodione**	0.99	112	102	*	20	*	20	0.99	114	71	*	17	20	20
**Isobenzan**	0.99	89	87	6	2	12	11	0.98	97	76	4	10	11	16
**Isocarbophos**	0.99	78	83	7	1	2	8	0.99	84	78	5	9	7	14
**Isodrin**	0.99	81	87	1	1	7	6	0.99	114	76	3	9	5	13
**Isofenphos**	0.99	96	94	2	1	8	6	0.99	120	76	7	8	20	10
**Isofenphos-methyl**	0.99	104	97	6	1	8	6	0.98	118	75	4	9	12	12
**Isomethiozin**	0.99	97	95	4	2	7	5	0.99	120	<70	7	10	9	16
**Isopropalin**	0.99	60	73	2	1	3	6	0.98	120	72	7	9	10	18
**Isoprothiolane**	0.99	88	89	1	1	5	4	0.98	119	78	3	11	6	13
**Kresoxim-methyl**	0.99	79	90	6	1	5	3	0.99	120	76	10	10	9	10
**Lambda Cyhalothrin**	0.99	80	88	5	1	14	20	0.99	>120	78	9	12	12	19

*acceptable value at 5 ppb

Table S2. (Continued)

Compound	APPLE							APPLE JUICE						
	Linearity (R^2)	Recovery		Precision				Linearity (R^2)	Recovery		Precision			
		R (%)		Intraday (%)		Interday (%)			R (%)		Intraday (%)		Interday (%)	
		1 µg kg^{-1}	10 µg kg^{-1}	1 µg kg^{-1}	10 µg kg^{-1}	1 µg kg^{-1}	10 µg kg^{-1}		1 µg L^{-1}	10 µg L^{-1}	1 µg L^{-1}	10 µg L^{-1}	1 µg L^{-1}	10 µg L^{-1}
**Lenacile**	0.99	103	89	7	2	16	8	0.99	93	<70	2	20	20	20
**Leptophos**	0.99	86	85	4	3	15	10	0.98	120	70	5	11	12	15
**Lindane-gamma**	0.99	97	98	1	1	13	3	0.98	119	83	1	10	4	20
**Malathion**	0.99	73	75	7	1	12	8	0.99	105	73	19	9	20	12
**Mefenpyr-diethyl**	0.99	78	92	6	2	*	6	0.99	120	74	9	9	16	11
**Mepanipyrim**	0.99	106	97	0	1	4	5	0.97	>120	76	9	10	6	12
**Metalaxyl**	0.99	89	87	3	1	11	4	0.99	120	81	17	8	10	17
**Metazachlor**	0.99	98	104	3	4	6	7	0.98	119	76	2	11	1	19
**Methamidophos**	0.99	76	79	*	14	8	20	0.97	92	<70	15	20	*	10
**Methidation**	0.99	97	96	6	2	20	18	0.99	112	74	8	8	10	10
**Methoxychlor**	0.99	87	94	2	1	20	17	0.98	118	72	4	12	18	20
**Metolachlor**	0.99	95	99	4	1	9	7	0.98	120	77	9	9	3	15
**Mevinphos**	0.99	91	94	2	1	19	16	0.99	120	80	2	9	2	18
**Mirex**	0.99	78	83	8	1	18	11	0.99	118	80	1	9	6	17
**Myclobutanil**	0.99	79	86	1	2	7	7	0.98	>120	74	9	13	7	15
**Napropamide**	0.99	87	115	4	1	9	3	0.99	120	75	4	9	10	15
**Nicotine**	0.99	91	111	5	1	9	10	0.99	<70	<70	*	8		11
**Nitrofen**	0.99	89	96	1	2	20	14	0.98	119	75	18	10	19	15
**Nitrothal isopropyl**	0.99	105	95	7	1	14	5	0.99	120	72	7	11	16	15
**Norflurazon**	0.99	103	107	9	2	19	20	0.98	>120	81	20	10	13	20
**Nuarimol**	0.99	80	74	2	1	7	4	0.99	118	70	4	11		16
**o,p'-DDD (Mitotane)**	0.99	80	94	6	1	19	11	0.99	102	71	3	11	11	16
**o,p'-DDE (2,4-DDE)**	0.99	<50	<50	3	1	3	3	0.99	102	77	0	8	3	17
**o,p'-DDT**	0.99	76	97	4	3	18	11	0.99	102	71	3	11	11	15
**Oxadiazone**	0.99	77	101	1	1	*	6	0.98	119	77	2	10	10	17
**Oxadixyl**	0.99	91	86	2	1	14	4	0.99	>120	74	7	12	*	12
**Oxyfluorfen**	0.99	86	90	12	1	18	7	0.99	120	71	7	8	20	18
**p,p´-DDD**	0.99	114	84	3	1	3	3	0.99	120	80	1	10	7	19
**p,p´-DDE**	0.99	112	93	1	1	7	9	0.99	101	74	3	10	4	12
**p,p´-DDT**	0.99	90	73	1	2	16	19	0.99	99	71	11	4	14	20
**Parathion**	0.99	110	90	9	2	20	17	0.99	117	72	7	9	12	16
**Parathion-methyl**	0.99	79	95	9	1	18	7	0.99	105	80	20	12	10	14
**Penconazole**	0.99	115	89	2	1	12	5	0.98	119	92	3	11	6	11
**Pendimethalin**	0.99	103	86	6	2	20	5	0.98	>120	70	2	10	14	10
**Pentachloroaniline**	0.99	79	104	1	1	8	2	0.99	118	74	3	9	7	12
**Pentachloroanisole**	0.99	96	80	6	1	16	5	0.99	103	72	6	10	2	19
**Pentachlorobenzene**	0.99	112	88	9	1	20	4	0.99	80	70	4	8	8	12

*acceptable value at 5 ppb

Table S2. (Continued)

Compound	APPLE							APPLE JUICE						
	Linearity (R^2)	Recovery		Precision				Linearity (R^2)	Recovery		Precision			
		R (%)		Intraday (%)		Interday (%)			R (%)		Intraday (%)		Interday (%)	
		1 µg kg^{-1}	10 µg kg^{-1}	1 µg kg^{-1}	10 µg kg^{-1}	1 µg kg^{-1}	10 µg kg^{-1}		1 µg L^{-1}	10 µg L^{-1}	1 µg L^{-1}	10 µg L^{-1}	1 µg L^{-1}	10 µg L^{-1}
**Permethrine**	0.99	75	107	19	2	19	13	0.98	>120	71	17	16	12	15
**Perthane**	0.98	107	86	12	3	18	10	0.98	120	74	6	10	7	15
**Phenisopham**	0.99	94	84	4	2	1	20	0.99	120	70	8	12	14	14
**Phenol 2,4,6-trichloro**	0.99	<50	<50	6	2	18	4	0.99	85	<70	9	8	15	10
**Phenthoate**	0.99	103	100	3	2	20	18	0.98	119	74	7	10	10	16
**Phosalone**	0.99	83	86	8	3	8	19	0.99	>120	70	11	11	17	16
**Phosmet**	0.99	92	85	5	2	18	20	0.98	119	70	10	13	4	20
**Piperonylbutoxide**	0.99	85	101	13	1	19	5	0.99	118	74	10	11	2	14
**Piperophos**	0.99	77	70	3	1	23	19	0.98	115	70	14	11	1	20
**Pirimicarb**	0.99	82	109	6	1	5	4	0.99	120	79	4	10	4	19
**Pirimiphos-ethyl**	0.99	80	81	7	1	5	5	0.99	119	76	4	11	14	17
**Pirimiphos-methyl**	0.99	114	111	1	2	10	4	0.99	121	74	1	10	12	10
**Plifenat**	0.99	84	78	*	1	18	4	0.98	119	71	3	13	20	14
**Prallethrin**	0.99	111	114	*	4	7	13	0.99	104	76	7	20	*	11
**Prochloraz**	0.99	101	112	*	1	20	15	0.99	>120	70	19	20	20	20
**Procymidone**	0.99	89	97	4	3	12	6	1.00	117	79	14	11	20	19
**Profenofos**	0.99	106	111	6	5	*	16	0.98	117	72	5	12	12	13
**Profluralin**	0.99	105	78	1	1	*	10	0.99	105	70	2	9	19	12
**Prometryn**	0.99	104	103	3	1	1	2	0.98	120	76	10	9	8	14
**Propachlor**	0.99	87	96	3	1	18	5	0.99	120	85	1	9	12	12
**Propanil**	0.99	84	101	4	1	20	17	0.99	120	76	5	9	14	10
**Propargite**	0.99	73	73	1	1	11	16	0.99	109	72	13	9	20	12
**Propham**	0.99	90	109	3	3	11	10	0.99	111	80	2	8	17	11
**Propiconazole**	0.99	76	85	2	1	18	5	0.99	119	86	7	20	12	19
**Propoxur**	0.99	114	82	2	1	19	20	0.98	120	80	4	10	2	12
**Propyzamide**	0.99	75	85	1	2	7	10	0.99	119	79	3	10	7	10
**Prothiofos**	0.99	70	100	1	2	16	18	0.97	119	75	1	10	2	10
**Pyrazophos**	0.99	92	80	*	4	16	20	0.99	>120	70	7	18	15	18
**Pyridaben**	0.99	95	75	*	16	5	8	0.99	>120	71	13	15	*	18
**Pyridalyl**	0.98	92	71	6	2	*	19	0.99	92	70	6	11	20	20
**Pyridaphenthion**	0.99	106	97	2	3	8	4	0.98	101	70	18	11	13	20
**Pyrifenox**	0.99	100	89	2	1	8	5	0.99	>120	71	8	10	11	18
**Pyrimethanil**	0.99	91	79	3	1	20	7	0.99	111	82	10	10	4	15
**Pyriproxyfen**	0.99	87	115	1	1	9	8	0.98	120	70	5	13	5	14
**Quinalphos**	0.99	75	79	2	1	8	3	0.98	119	74	9	10	13	11
**Quinoxyfen**	0.99	111	95	2	1	13	4	0.99	120	73	7	8	3	16
**Quintozene**	0.99	112	115	5	2	12	6	0.99	103	72	6	10	2	19

*acceptable value at 5 ppb

Table S2. (Continued)

Compound	APPLE							APPLE JUICE						
	Linearity (R^2)	Recovery		Precision				Linearity (R^2)	Recovery		Precision			
		R (%)		Intraday (%)		Interday (%)			R (%)		Intraday (%)		Interday (%)	
		1 µg kg^{-1}	10 µg kg^{-1}	1 µg kg^{-1}	10 µg kg^{-1}	1 µg kg^{-1}	10 µg kg^{-1}		1 µg L^{-1}	10 µg L^{-1}	1 µg L^{-1}	10 µg L^{-1}	1 µg L^{-1}	10 µg L^{-1}
**Resmethrin**	0.99	111	79	12	3	17	12	0.98	120	72	3	7	19	20
**S421 (Octachlorodipropyl ether)**	0.99	79	86	11	1	16	18	0.99	112	71	3	4	8	16
**Silafluorfen**	0.99	99	92	20	2	20	13	0.98	>120	83	67	12	15	12
**Simazina**	0.99	102	85	5	3	15	10	0.99	116	80	5	9	18	20
**Spiromesifen**	0.99	91	111	6	1	9	6	0.98	>120	70	7	10	13	16
**Spiroxamine**	0.99	89	95	4	1	6	5	0.98	>120	96	17	11	16	16
**Sulfotep**	0.99	101	114	2	1	20	10	0.98	111	75	6	11	12	18
**Sulprofos**	0.99	76	103	4	2	18	8	0.98	>120	79	5	10	8	15
**Tebuconazole**	0.99	113	70	9	7	15	10	0.98	118	73	8	9	13	15
**Tebufenpyrad**	0.99	73	89	20	2	9	5	0.98	>120	74	3	13	20	16
**Tecnazene**	0.99	96	88	5	3	1	3	0.98	95	75	3	12	2	18
**Tefluthrine**	0.99	86	110	2	1	1	5	0.99	113	78	1	9	6	19
**Terbacil**	0.99	77	96	3	1	20	16	0.99	120	71	7	10	6	14
**Terbufos**	0.99	104	74	20	20	18	3	0.99	>120	85	11	6	11	10
**Terbumeton**	0.99	107	110	5	1	5	5	0.99	118	78	7	11	2	14
**Terbuthylazine**	0.99	91	110	4	1	5	4	0.98	120	79	3	9	4	10
**Terbutryn**	0.99	105	74	6	1	14	16	0.99	>120	74	4	10	10	12
**Tetrachlorvinphos**	0.99	92	107	3	4	14	5	0.98	>120	70	12	12	6	16
**Tetraconazole**	0.99	112	70	7	1	15	7	0.98	119	71	11	10	7	18
**Tetradiphon**	0.99	103	97	19	3	20	6	0.99	90	75	2	9	*	15
**Tetramethrin**	0.99	109	97	20	5	31	3	0.98	>120	71	14	37	5	18
**Tetrasul**	0.99	94	104	2	1	6	4	0.99	121	75	3	9	12	17
**Thiobencarb**	0.99	83	108	6	1	19	9	0.99	120	73	4	9	9	12
**Thiofanox**	0.99	95	88	20	1	16	6	0.99	76	43	*	20	20	13
**Thiometon**	0.99	90	78	4	3	6	6	0.99	<70	>120	*	9	*	14
**Tolclofos-methyl**	0.99	81	93	3	1	14	11	0.99	117	76	8	10	5	12
**Tolylfluanid**	0.99	107	93	5	1	5	4	0.99	82	58	3	10	12	15
**Transfluthrin**	0.99	83	108	4	1	7	2	0.99	120	75	9	11	5	10
**Triadimefon**	0.99	90	88	9	2	12	7	0.98	120	82	8	8	2	18
**Triadimenol**	0.99	75	115	6	3	18	16	0.98	118	80	10	9	16	19
**Triazophos**	0.99	107	107	6	20	13	8	0.99	112	74	11	12	*	14
**Trichloronat**	0.99	81	102	3	1	14	20	0.99	116	72	2	10	5	14
**Trifloxystrobin**	0.99	82	92	11	1	6	8	0.98	111	77	20	12	13	20
**Trifluralin**	0.99	93	109	4	1	8	5	0.98	118	72	20	10	8	15
**Vinclozoline**	0.99	106	93	1	1	7	20	0.99	120	76	1	10	20	18
**Zoxamida**	0.99	83	101	8	3	6	5	0.99	>120	70	20	8	*	15

*acceptable value at 5 ppb

Table S3. Limits of detection (LODs) and quantification (LOQs) for the validation in apple and apple juice.

Compound	APPLE			APPLE JUICE		
	LOD (μg kg-1)	LOQ (μg kg-1)		LOD (μg L-1)	LOQ (μg L-1)	
		1*	2*		1*	2*
**1.4-dimethylnaphthalene**	0.10	0.34	0.50	0.06	0.19	0.50
**2-phenylphenol**	0.21	0.70	0.50	0.15	0.49	0.50
**3.5-Dichloroaniline**	0.30	1.00	0.50	0.12	0.39	0.50
**4.4Dibromobenzophenone**	0.25	0.82	0.50	0.23	0.78	0.50
**4.4-Dichlorobenzophenone**	0.30	0.99	0.50	0.13	0.44	0.50
**4-Chloro-3-methylphenol**	0.10	0.33	0.50	1.05	3.51	0.50
**Aclonifen**	0.42	1.40	1.00	0.14	0.47	1.00
**Acrinathrin**	0.57	1.89	1.00	0.62	2.06	5.00
**Alachlor**	0.05	0.17	0.50	0.05	0.17	0.50
**Aldrin**	0.16	0.55	0.50	0.07	0.24	0.50
**Anthraquinone**	0.35	1.16	0.50	0.16	0.54	0.50
**Atrazine**	0.05	0.18	0.50	0.41	1.37	0.50
**Azoxystrobin**	0.43	1.44	1.00	0.05	0.17	0.50
**Benalaxyl**	0.11	0.36	0.50	0.19	0.64	1.00
**Benfluralin**	0.26	0.88	0.50	0.08	0.25	0.50
**Benfuresate**	0.06	0.21	0.50	0.10	0.35	0.50
**Benodalil**	0.08	2.16	0.50	0.05	0.15	0.50
**Benoxacor**	0.22	0.74	0.50	0.06	0.19	0.50
**Benzene. hexachloro**	0.18	0.60	0.50	0.28	0.94	0.50
**Benzyl benzoate**	0.31	1.03	0.50	0.02	0.08	0.50
**Bifenazato**	0.74	2.47	1.00	1.30	4.32	0.50
**Bifenox**	0.05	0.18	0.50	0.42	1.41	0.50
**Bifenthrin**	1.42	3.73	1.00	0.72	2.41	1.00
**Biphenyl**	0.29	0.97	0.50	0.23	0.76	0.50
**Bitertanol**	0.26	0.85	0.50	0.22	0.73	0.50
**Boscalid**	0.41	1.37	0.50	0.10	0.33	0.50
**Bromacil**	0.11	0.37	0.50	0.20	0.66	0.50
**Bromocyclen**	0.03	0.10	0.50	0.10	0.32	0.50
**Bromophos-ethyl**	0.17	0.55	0.50	0.18	0.59	0.50
**Bromophos-methyl**	0.20	0.65	0.50	0.10	0.35	0.50
**Bromopropylate**	0.09	0.28	0.50	0.24	0.82	0.50
**Bupirimate**	0.17	0.58	0.50	0.30	1.01	0.50
**Buprofezin**	0.10	0.32	0.50	0.13	0.42	0.50
**Butafenacil**	0.03	0.10	0.50	0.14	0.45	0.50
**Butilate**	0.77	0.90	1.00	0.37	1.22	1.00
**Butralin**	0.47	0.43	0.50	0.05	0.15	0.50
**Cadusafos**	0.08	0.26	0.50	0.14	0.46	0.50

**Carbophenothion**	0.24	0.79	0.50	0.07	0.23	0.50
**Carbophenothion methyl**	0.88	3.35	1.00	0.12	0.40	1.00
**Chinomethionate**	0.08	0.28	0.50	0.39	1.29	1.00
**Chlordane (CIS+TRANS)**	0.36	1.19	1.00	0.25	0.83	0.50
**Chlorfenapyr**	0.73	2.42	0.50	0.43	1.44	1.00
**Chlorfenprop methyl**	0.06	0.21	0.50	0.32	1.07	1.00
**Chlorfenson**	0.06	0.19	0.50	0.13	0.43	0.50
**Chlorfenvinphos**	0.07	0.23	0.50	0.21	0.69	0.50
**Chlorflurenol-methyl**	0.15	0.52	0.50	0.09	0.31	0.50
**Chlormephos**	0.15	0.49	0.50	0.10	0.33	0.50
**Chloropropylate**	0.74	0.96	0.50	0.20	0.67	0.50
**Chlorothalonil**	0.17	0.57	0.50	1.22	0.80	0.50
**Chlorpropham**	0.23	0.76	0.50	0.07	0.22	0.50
**Chlorpyrifos – ethyl**	0.16	0.53	0.50	0.11	0.37	0.50

1* Value of 10 times the standard deviations obtained for the lowest level of calibration curve. 2* Valoe of the lowest concentration level offering relative standard deviation (RSD) ≤ 20%.

Table S3. (Continued)

Compound	APPLE			APPLE JUICE		
	LOD (μg kg^{-1})	LOQ (μg kg^{-1})		LOD (μg/L)	LOQ (μg L^{-1})	
		1*	2*		1*	2*
**Chlorpyrifos – methyl**	0.20	0.68	0.50	0.11	0.37	0.50
**Chlorthion**	0.12	0.40	0.50	0.08	0.27	0.50
**Chlozolinate**	0.27	0.88	0.50	0.16	0.55	0.50
**Cinidon-ethyl**	0.19	1.29	0.50	0.37	1.22	1.00
**Clodinafop-propargyl**	0.91	3.27	1.00	0.23	0.77	0.50
**Crimidine**	0.14	0.46	0.50	0.16	0.52	0.50
**Cyanofenphos**	0.24	0.79	0.50	0.27	0.91	0.50
**Cyanophos**	0.19	0.62	0.50	0.06	0.20	0.50
**Cycloate**	0.24	0.82	0.50	0.08	0.27	0.50
**Cyflufenamid**	0.14	0.45	1.00	0.34	1.15	1.00
**Cyfluthrin**	0.21	0.71	1.00	0.16	0.52	1.00
**Cypermethrin**	1.08	3.60	1.00	1.80	5.99	0.50
**Cyproconazole**	0.22	0.75	0.50	0.19	0.63	0.50
**Cyprodinil**	0.09	0.29	0.50	0.32	1.06	0.50
**DCPA**	0.07	0.23	0.50	0.25	0.85	0.50
**Deltamethrin**	0.42	1.41	0.50	0.43	2.76	1.00
**Diazinone**	0.10	0.34	0.50	0.11	0.35	0.50
**Dichlobenil**	0.12	0.42	0.50	0.07	0.24	0.50
**Dichlofenthion**	0.19	0.62	0.50	0.09	0.31	0.50
**Dichloran**	0.21	0.71	0.50	0.36	1.21	1.00
**Dichlorvos**	0.04	0.13	0.50	0.09	0.31	0.50
**Diclofop-methyl**	0.31	1.04	1.00	0.42	1.39	1.00
**Dicofol. 4.4**	0.74	2.45	0.50	0.20	0.67	0.50

**Dieldrin**	0.39	1.29	0.50	0.55	1.85	0.50
**Difenoconazole**	0.92	3.06	0.50	0.75	2.49	5.00
**Diflufenican**	0.17	0.58	0.50	0.13	0.43	0.50
**Dimethomorph**	0.75	3.30	1.00	1.01	3.36	5.00
**Diniconazole**	0.07	0.24	0.50	0.02	0.06	5.00
**Diphenylamine**	0.19	0.63	0.50	0.28	0.92	0.50
**Disulfoton**	0.25	0.84	0.50	0.11	0.37	1.00
**Ditalimfos**	0.20	0.67	0.50	0.17	0.55	0.50
**Edifenphos**	0.15	0.51	0.50	0.08	0.28	0.50
**Endosulfan alpha**	0.03	0.10	0.50	0.10	0.34	1.00
**Endosulfan beta**	0.28	0.94	1.00	0.18	0.61	1.00
**Endosulfan ether**	0.09	0.29	0.50	0.49	1.62	0.50
**Endosulfan sulfate**	0.19	0.62	0.50	0.61	2.03	1.00
**Endrin**	0.39	1.29	0.50	0.01	0.05	0.50
**Endrin ketone**	0.34	1.13	1.0	0.03	0.09	0.50
**EPTC (Eptam)**	0.10	0.35	0.50	0.21	0.69	0.50
**Esfenvalerate**	0.11	0.35	0.50	0.22	0.73	0.50
**Ethion**	0.10	0.34	0.50	0.30	0.98	5.00
**Ethoprophos**	0.13	0.44	0.50	0.20	0.67	0.50
**Ethoxyquin**	0.48	1.75	0.50	0.33	1.11	0.50
**Etridiazole**	0.36	1.19	0.50	0.12	0.40	1.00
**Etrimfos**	0.20	0.65	0.50	0.18	0.60	1.00
**Famfur (Fonofos)**	0.23	0.76	0.50	0.10	0.34	5.00
**Fenamiphos**	0.79	2.63	1.00	0.99	3.29	5.00
**Fenamiphos sulfone**	0.45	1.49	0.50	0.48	1.61	1.00
**Fenamiphos sulfoxide**	0.38	1.39	0.50	0.63	2.09	5.00
**Fenarimol**	0.14	0.46	0.50	0.50	1.67	1.00
**Fenazaquin**	0.57	2.24	1.00	0.05	0.18	0.50

1* Value of 10 times the standard deviations obtained for the lowest level of calibration curve. 2* Valoe of the lowest concentration level offering relative standard deviation (RSD) ≤ 20%.

Table S3. (Continued)

Compound	APPLE			APPLE JUICE		
	LOD (μg kg^{-1})	LOQ (μg kg^{-1})		LOD (μg L^{-1})	LOQ (μg L^{-1})	
		1*	2*		1*	2*
**Fenbuconazol**	0.15	0.49	0.50	0.79	2.64	1.00
**Fenchlorphos/Ronnel**	0.11	0.38	0.50	0.05	0.18	0.50
**Fenhexamid**	0.27	1.55	0.50	0.76	2.52	0.50
**Fenitrothion**	0.28	0.94	0.50	0.70	2.33	1.00
**Fenobucarb**	0.02	0.05	0.50	0.33	1.11	1.00
**Fenoxaprop-P-ethyl**	0.16	0.53	0.50	0.53	1.77	1.00
**Fenoxycarb**	0.16	3.88	0.50	0.10	0.32	0.50
**Fenpropathrin**	0.05	0.18	0.50	0.35	1.17	1.00
**Fenpropimorph**	0.09	0.31	0.50	0.04	0.12	0.50
**Fenson**	0.67	3.80	0.50	0.03	0.11	5.00
**Fenthion**	0.32	1.08	0.50	0.11	0.36	0.50
**Fenvalerate**	0.56	1.86	1.00	0.16	0.53	1.00
**Fipronil**	0.31	1.04	0.50	0.88	2.93	1.00

**Fipronil sulfone**	0.14	0.46	0.50	0.24	0.81	0.50
**Fluchloralin**	0.27	0.89	0.50	0.55	1.83	0.50
**Flucythrinate**	0.41	2.70	0.50	0.08	0.27	0.50
**Fludioxonil**	1.63	5.44	0.50	0.08	0.27	0.50
**Flumetralin**	0.19	0.63	0.50	0.34	1.15	1.00
**Flumioxazin**	0.88	2.92	0.50	0.65	2.15	1.00
**Fluotrimazole**	0.06	0.21	0.50	0.19	0.62	1.00
**Fluquinconazole**	0.43	1.44	0.50	0.56	1.85	1.00
**Fluvalinate (Tau)**	0.23	0.76	1.00	0.13	0.42	1.00
**Fonofos**	0.29	0.98	0.50	0.03	0.11	0.50
**Formothion**	0.09	0.31	0.50	0.14	0.47	0.50
**Furalaxyl**	0.17	0.57	0.50	0.20	0.67	0.50
**Halfenprox**	0.05	0.16	0.50	0.51	1.70	1.00
**Heptachlor**	0.21	0.70	0.50	0.28	0.95	0.50
**Heptachlor-epoxide-A-endo (cis)**	0.30	1.00	0.50	0.07	0.24	0.50
**Heptachlor-epoxide-A-endo (trans)**	0.15	0.51	0.50	0.11	0.37	0.50
**Heptenophos**	0.30	1.00	1.00	0.14	0.47	1.00
**Hexachlorocychlohexane-Alpha**	0.28	0.94	0.50	0.34	2.14	1.00
**Hexachlorocyclohexano-Beta**	0.13	0.44	0.50	0.25	0.85	0.50
**Hexachlorocyclohexano-Delta**	0.06	0.22	0.50	1.16	3.87	1.00
**Hexaconazole**	0.69	2.31	1.00	0.27	0.90	5.00
**Hexazinone**	0.23	0.78	0.50	1.44	4.81	1.00
**Indoxacarb**	0.31	1.05	1.00	0.17	0.55	5.00
**Iodofenphos**	0.25	0.83	0.50	0.12	0.39	0.50
**Iprodione**	0.50	2.99	1.00	0.08	0.28	5.00
**Isobenzan**	0.28	0.94	0.50	0.23	0.76	1.00
**Isocarbophos**	0.48	1.61	0.50	0.22	0.74	0.50
**Isodrin**	0.03	0.10	0.50	0.14	0.47	0.50
**Isofenphos**	0.05	0.16	0.50	0.22	0.74	0.50
**Isofenphos-methyl**	0.24	0.81	0.50	0.09	0.29	0.50
**Isomethiozin**	0.31	1.03	0.50	0.18	0.61	0.50
**Isopropalin**	0.15	0.51	0.50	0.26	0.88	0.50
**Isoprothiolane**	0.14	0.46	0.50	0.58	1.93	1.00
**Kresoxim-methyl**	0.09	0.31	0.50	0.20	0.65	0.50
**Lambda Cyhalothrin**	0.50	1.66	0.50	0.13	0.44	0.50
**Lenacile**	0.12	0.42	0.50	0.24	0.79	1.00
**Leptophos**	0.09	0.31	0.50	0.21	0.71	0.50

1* Value of 10 times the standard deviations obtained for the lowest level of calibration curve. 2* Valoe of the lowest concentration level offering relative standard deviation (RSD) ≤ 20%.

Table S3. (Continued)

Compound	APPLE			APPLE JUICE		
	LOD (μg kg^{-1})	LOQ (μg kg^{-1})		LOD (μg L^{-1})	LOQ (μg L^{-1})	
		1*	2*		1*	2*
**Lindane-gamma**	0.28	0.94	0.50	0.14	0.46	0.50
**Malathion**	0.18	0.61	0.50	0.14	0.45	0.50
**Mefenpyr-diethyl**	0.31	1.03	0.50	0.02	0.06	0.50
**Mepanipyrim**	0.18	0.61	0.50	1.74	5.80	1.00
**Metalaxyl**	0.08	0.27	0.50	0.16	0.52	0.50
**Metazachlor**	0.36	1.20	0.50	0.42	1.39	1.00
**Methamidophos**	0.53	1.76	1.00	0.06	0.21	0.50

**Methidation**	0.30	0.99	0.50	0.03	0.11	0.50
**Methoxychlor**	0.21	0.72	0.50	0.10	0.33	0.50
**Metolachlor**	0.09	0.30	0.50	0.12	0.42	0.50
**Mevinphos**	0.05	0.16	0.50	0.19	0.63	0.50
**Mirex**	0.17	0.57	0.50	0.20	0.65	1.00
**Myclobutanil**	0.04	0.12	0.50	0.51	1.70	1.00
**Napropamide**	0.35	1.17	0.50	0.30	0.99	0.50
**Nicotine**	0.13	0.42	0.50	0.05	0.15	5.00
**Nitrofen**	0.34	1.14	0.50	0.48	1.61	1.00
**Nitrothal isopropyl**	0.11	0.36	0.50	0.05	0.17	0.50
**Norflurazon**	0.41	1.38	0.50	0.11	0.38	0.50
**Nuarimol**	0.04	0.14	0.50	0.16	0.54	0.50
**o.p'-DDD (Mitotane)**	0.21	0.69	0.50	0.36	1.20	1.00
**o.p'-DDE (2.4-DDE)**	0.25	0.83	0.50	0.44	1.47	0.50
**o.p'-DDT**	0.56	1.86	0.50	0.11	0.38	0.50
**Oxadiazone**	0.26	0.86	0.50	0.32	1.05	0.50
**Oxadixyl**	0.05	0.17	0.50	0.25	0.85	0.50
**Oxyfluorfen**	0.22	0.75	0.50	0.65	2.15	1.00
**p.p´-DDD**	0.02	0.08	0.50	0.19	0.62	1.00
**p.p´-DDE**	0.25	0.83	0.50	0.56	1.85	1.00
**p.p´-DDT**	0.59	1.98	0.50	0.13	0.42	1.00
**Parathion**	0.27	0.89	0.50	0.23	0.75	0.50
**Parathion-methyl**	0.21	0.70	0.50	0.05	0.16	0.50
**Penconazole**	0.04	0.14	0.50	0.11	0.37	0.50
**Pendimethalin**	0.36	1.20	0.50	0.20	0.67	0.50
**Pentachloroaniline**	0.05	0.16	0.50	0.10	0.33	0.50
**Pentachloroanisole**	0.16	0.53	0.50	0.03	0.09	0.50
**Pentachlorobenzene**	0.43	1.45	0.50	0.08	0.28	0.50
**Permethrine**	0.42	1.41	1.00	0.19	0.63	0.50
**Perthane**	1.03	3.11	1.00	0.14	0.48	0.50
**Phenisopham**	0.32	1.06	0.50	0.13	0.42	0.50
**Phenol 2.4.6-trichloro**	0.11	0.38	0.50	0.25	0.82	0.50
**Phenthoate**	0.31	1.05	0.50	0.31	1.03	0.50
**Phosalone**	0.33	1.11	0.50	0.21	0.70	0.50
**Phosmet**	0.71	2.37	0.50	0.08	0.27	0.50
**Piperonylbutoxide**	0.32	1.06	0.50	0.04	0.13	0.50
**Piperophos**	0.11	0.38	0.50	0.45	1.51	0.50
**Pirimicarb**	1.78	5.93	0.50	0.15	0.49	0.50
**Pirimiphos-ethyl**	0.14	0.47	0.50	0.26	0.85	0.50
**Pirimiphos-methyl**	0.14	0.48	0.50	0.20	0.67	0.50
**Plifenat**	0.46	1.54	0.50	0.70	2.35	1.00
**Prallethrin**	0.32	1.08	1.00	0.79	2.63	1.00
**Prochloraz**	0.64	2.14	1.00	0.23	0.77	1.00

Procymidone	1.01	3.35	1.00	0.28	0.95	1.00

1* Value of 10 times the standard deviations obtained for the lowest level of calibration curve. 2* Valoe of the lowest concentration level offering relative standard deviation (RSD) ≤ 20%.

Table S3. (Continued)

Compound	APPLE			APPLE JUICE		
	LOD (μg kg^{-1})	LOQ (μg kg^{-1})		LOD (μg L^{-1})	LOQ (μg L^{-1})	
		1*	2*		1*	2*
**Profenofos**	0.09	0.29	0.50	0.43	1.42	1.00
**Profluralin**	0.38	1.25	0.50	0.69	2.30	1.00
**Prometryn**	0.04	0.13	0.50	0.14	0.47	0.50
**Propachlor**	0.05	0.15	0.50	0.21	0.69	0.50
**Propanil**	0.36	1.21	0.50	0.18	0.60	0.50
**Propargite**	0.21	0.71	0.50	0.37	1.23	1.00
**Propham**	0.29	0.96	0.50	0.23	0.78	0.50
**Propiconazole**	0.10	0.35	0.50	0.24	0.78	0.50
**Propoxur**	0.21	0.70	0.50	0.75	2.50	0.50
**Propyzamide**	0.11	0.35	0.50	0.12	0.39	0.50
**Prothiofos**	0.02	0.07	0.50	0.04	0.15	0.50
**Pyrazophos**	0.42	1.39	0.50	0.29	0.96	1.00
**Pyridaben**	0.43	1.44	1.00	0.73	2.44	1.00
**Pyridalyl**	1.05	3.49	1.00	0.17	0.55	1.00
**Pyridaphenthion**	0.39	1.30	0.50	0.46	1.54	1.00
**Pyrifenox**	0.27	0.91	0.50	0.30	1.00	0.50
**Pyrimethanil**	0.06	0.20	0.50	0.12	0.40	0.50
**Pyriproxyfen**	0.37	1.25	0.50	0.08	0.27	0.50
**Quinalphos**	0.16	0.52	0.50	0.22	0.72	0.50
**Quinoxyfen**	0.19	0.63	0.50	0.06	0.19	0.50
**Quintozene**	0.23	0.78	0.50	0.03	0.09	0.50
**Resmethrin**	1.33	4.43	0.50	1.56	5.20	0.50
**S421 (Octachlorodipropyl ether)**	0.14	0.46	0.50	0.14	0.45	0.50
**Silafluorfen**	0.03	0.09	1.00	0.32	1.07	1.00
**Simazina**	0.12	0.38	0.50	2.89	9.64	1.00
**Spiromesifen**	0.15	0.49	0.50	0.24	0.81	0.50
**Spiroxamine**	0.13	0.42	0.50	0.22	0.75	0.50
**Sulfotep**	0.05	0.15	0.50	0.22	0.73	0.50
**Sulprofos**	0.29	0.98	0.50	0.10	0.34	0.50
**Tebuconazole**	0.16	0.55	0.50	0.52	1.74	1.00
**Tebufenpyrad**	0.91	3.04	1.00	0.11	0.35	1.00
**Tecnazene**	0.13	0.45	0.50	0.03	0.10	0.50
**Tefluthrine**	0.14	0.46	0.50	0.10	0.33	0.50
**Terbacil**	0.07	0.23	0.50	0.07	0.25	0.50
**Terbufos**	0.95	2.49	1.00	0.41	1.37	1.00
**Terbumeton**	0.14	0.47	0.50	0.04	0.14	0.50

**Terbuthylazine**	0.10	0.35	0.50	0.13	0.44	0.50
**Terbutryn**	0.14	0.47	0.50	0.18	0.59	0.50
**Tetrachlorvinphos**	0.34	1.12	0.50	0.10	0.34	0.50
**Tetraconazole**	0.05	0.17	0.50	0.17	0.57	0.50
**Tetradiphon**	0.52	1.72	1.00	0.65	2.16	1.00
**Tetramethrin**	0.86	2.88	1.00	0.65	2.17	0.50
**Tetrasul**	0.05	0.17	0.50	0.20	0.66	0.50
**Thiobencarb**	0.14	0.47	0.50	0.16	0.53	0.50
**Thiofanox**	0.78	2.59	1.00	1.32	4.38	1.00
**Thiometon**	0.50	1.68	0.50	0.05	0.17	1.00
**Tolclofos-methyl**	0.16	0.54	0.50	0.08	0.28	0.50
**Tolylfluanid**	0.08	0.27	0.50	0.20	0.68	5.00
**Transfluthrin**	0.10	0.34	0.50	0.07	0.24	5.00
**Triadimefon**	0.14	0.46	0.50	0.03	0.10	0.50

1* Value of 10 times the standard deviations obtained for the lowest level of calibration curve. 2* Valoe of the lowest concentration level offering relative standard deviation (RSD) ≤ 20%.

Table S3. (Continued)

Compound	APPLE			APPLE JUICE		
	LOD (μg kg^{-1})	LOQ (μg kg^{-1})		LOD (μg L^{-1})	LOQ (μg L^{-1})	
		1*	2*		1*	2*
**Triadimenol**	0.44	2.12	0.50	0.24	0.79	0.50
**Triazophos**	0.20	2.01	0.50	0.97	3.24	1.00
**Trichloronat**	0.06	0.20	0.50	0.10	0.33	0.50
**Trifloxystrobin**	0.45	2.16	0.50	0.05	0.15	0.50
**Trifluralin**	0.19	0.62	0.50	0.10	0.35	0.50
**Vinclozoline**	0.03	0.10	0.50	0.29	0.98	0.50
**Zoxamida**	0.16	0.53	0.50	0.80	1.32	1.00

1* Value of 10 times the standard deviations obtained for the lowest level of calibration curve. 2* Value of the lowest concentration level offering relative standard deviation (RSD) ≤ 20%.

Table S4. List of suspected compounds searched by retrospective analysis

Matrix	Compound	Elemental Composition	Most abundant fragment (*m*/*z*)	Reference
Juice	Acetochlor	$C_{14}H_{20}ClNO_2$	59.04968	[43]
Juice	Pretilachlor	$C_{17}H_{26}ClNO_2$	162.03218	[20]
Juice and apple	Azinphos-ethyl	$C_{12}H_{16}N_3O_3PS_2$	132.01978	[21]
Juice and apple	Azinphos-methyl	$C_{10}PN_3H_{12}S_2O_3$	160.05108	
Juice and apple	Carbendazim	$C_9H_9N_3O_2$	159.04326	
Juice and apple	Dimethoate	$C_5H_{12}NO_3PS_2$	87.01427	
Juice and apple	Dithiocarbamates	$CH_2NS_2^-$	81.03404	

Juice and apple	Fluazinam	$C_{13}H_4Cl_2F_6N_4O_4$	375.95213	
Juice and apple	Folpet	$C_9H_4Cl_3NO_2S$	259.93398	
Juice	Flusilazole	$C_{16}H_{15}F_2N_3Si$	233.05981	[18]
Juice	Pyraclostrobin	$C_{19}H_{18}ClN_3O_4$	132.04494	
Juice	Spirodiclofen	$C_{21}H_{24}Cl_2O_4$	71.08608	
Juice	Phorate	$C_7H_{17}O_2PS_3$	75.02685	
Juice	Fluopyram	$C_{16}H_{11}ClF_6N_2O$	44.0136	
Apple	naled	$C_4H_7Br_2Cl_2O_4P$	109.00545	[44]
Apple	Oxamyl	$C_7H_{13}N_3O_3S$	72.04494	
Apple	Imazalil	$C_{14}H_{14}Cl_2N_2O$	41.03913	
Apple	cymoxanil	$C_7H_{10}N_4O_3$	44.050024	
Apple	Azaconazole	$C_{12}H_{11}Cl_2N_3O_2$	216.98231	
Apple	cycluron	$C_{11}H_{22}N_2O$	72.04494	
Juice	Triticonazole	$C_{17}H_{20}ClN_3O$	235.08897	[45]
Juice	Phthalimide	$C_8H_5NO_2$	147.03203	[35]
Juice	Captan	$C_9H_8Cl_3NO_2S$	79.05477	
Juice	captafol	$C_{10}H_9Cl_4NO_2S$	79.05477	
Juice	Tetrahydrophthalimide	$C_8H_9NO_2$	79.05477	

Table S5. Summary of data obtained for validation tests performed to tetrahydrophthalimide and phthalimide

Compound	APPLE							APPLE JUICE						
	Linearity (R^2)	Recovery		Precision				Linearity (R^2)	Recovery		Precision			
		R (%)		Intraday (%)		Interday (%)			R (%)		Intraday (%)		Interday (%)	
		1 µg kg^{-1}	10 µg kg^{-1}	1 µg kg^{-1}	10 µg kg^{-1}	1 µg kg^{-1}	10 µg kg^{-1}		1 µg L^{-1}	10 µg L^{-1}	1 µg L^{-1}	10 µg L^{-1}	1 µg L^{-1}	10 µg L^{-1}
**Tetrahydrophthalimide**	0.99	85	89	*	2	3	1	0.98	103	79	4	11	16	15
**Phthalimide**	0.99	106	90	10	2	6	20	0.99	120	78	4	9	14	12

*acceptable value at 5 ppb

Table S6. Limits of detection (LODs) and quantification (LOQs) for the validation of tetrahydrophtalimide and phthalimide in apple and apple juice.

Compound	APPLE			APPLE JUICE		
	LOD (µg kg^{-1})	LOQ (µg kg^{-1})		LOD (µg L^{-1})	LOQ (µg L^{-1})	
		1*	2*		1*	2*
**Tetrahydrophthalimide**	0.33	1.09	1.00	0.73	3.77	1.00
**Phthalimide**	0.41	1.35	0.50	0.24	0.80	0.50

1* Value of 10 times the standard deviations obtained for the lowest level of calibration curve. 2* Valoe of the lowest concentration level offering relative standard deviation (RSD) ≤ 20%.
